# MoZn-based high entropy alloy catalysts enabled dual activation and stabilization in alkaline oxygen evolution

**DOI:** 10.1126/sciadv.adq6758

**Published:** 2024-11-20

**Authors:** Yunjie Mei, Jinli Chen, Qi Wang, Yaqing Guo, Hanwen Liu, Wenhui Shi, Cheng Lin, Yifei Yuan, Yuhua Wang, Bao Yu Xia, Yonggang Yao

**Affiliations:** ^1^ State Key Laboratory of Materials Processing and Die & Mould Technology, School of Materials Science and Engineering, Huazhong University of Science and Technology, Wuhan 430074 China.; ^2^ Science and Technology on Surface Physics and Chemistry Laboratory, Mianyang, Sichuan 621908, China.; ^3^ College of Chemistry and Materials Engineering, Wenzhou University, Wenzhou, China.; ^4^ Hubei Province Key Laboratory of Science in Metallurgical Process, Wuhan University of Science and Technology, Wuhan 430081, China.; ^5^ Key Laboratory of Material Chemistry for Energy Conversion and Storage (Ministry of Education), School of Chemistry and Chemical Engineering, Huazhong University of Science and Technology, Wuhan 430074, China.

## Abstract

It remains a grand challenge to develop electrocatalysts with simultaneously high activity, long durability, and low cost for the oxygen evolution reaction (OER), originating from two competing reaction pathways and often trade-off performances. The adsorbed evolution mechanism (AEM) suffers from sluggish kinetics due to a linear scaling relationship, while the lattice oxygen mechanism (LOM) causes unstable structures due to lattice oxygen escape. We propose a MoZnFeCoNi high-entropy alloy (HEA) incorporating AEM-promoter Mo and LOM-active Zn to achieve dual activation and stabilization for efficient and durable OER. Density functional theory and chemical probe experiments confirmed dual-mechanism activation, with representative Co-Co^†^-Mo sites facilitating AEM and Zn-O^†^-Ni sites enhancing LOM, resulting in an ultralow OER overpotential (η_10_ = 221 mV). The multielement interaction, high-entropy structure, and carbon network notably enhance structural stability for durable catalysis (>1500 hours at 100 mA cm^−2^). Our work offers a viable approach to concurrently enhance OER activity and stability by designing HEA catalysts to enable dual-mechanism synergy.

## Teaser

High-entropy design enables dual-mechanism activation and stabilization toward highly active and durable oxygen evolution.

## INTRODUCTION

Electrochemical water splitting requires highly active, long-durable, and cost-effective catalysts to meet the increasing needs for large-scale green hydrogen production and renewable energy storage, particularly for the sluggish and corrosive oxygen evolution reaction (OER). Co-, Ni-, and Fe-based catalysts have been considered as potential candidates to replace noble metals due to their tunable 3d electron configuration and spin state, versatility in terms of crystal and electronic structures, as well as abundance in nature (*1*, *2*). However, owing to the highly oxidative and corrosive conditions, Co-, Ni-, and Fe-based alloy catalysts undergo destructive reconstruction and are prone to erosion during long-term OER, thus jeopardizing their practical implementation (*3*, *4*). Thus far, it remains a grand challenge to develop advanced OER electrocatalysts with simultaneously high activity and long-term durability.

In electrochemical OER, there are two major reaction mechanisms as guiding principles for catalyst development. Generally, OER proceeds on Co-, Ni-, and Fe-based alloys via four concerted proton-electron transfer steps, also known as the adsorbate evolution mechanism (AEM) (*5*, *6*). Hence, the OER activity is mainly influenced by the adsorption energetics of the oxygen intermediates (O*, OH*, and OOH*) on the catalysts, which follow the Sabatier principle and the linear scaling relation (LSR), rendering a minimum theoretical overpotential of ~370 mV even for the best possible material (*7*). A common strategy to promote the AEM pathway is to regulate the adsorption energetics of the oxygen intermediates by alloying FeCoNi with oxyphilic elements such as Mo, among others (*6*). On the other hand, the lattice-oxygen–mediated mechanism (LOM) occurs via the O─O coupling of O* with lattice oxygen of oxides and does not involve the *OOH intermediate species, which overcomes the LSR constraint and results in much better theoretical OER activity (*8*). For instance, Zn was incorporated to partially replace Co in CoOOH to activate the lattice oxygen by weakening the Co─O bonds. Nevertheless, the resultant surface oxygen vacancies in the catalysts can lead to severe structural reconstruction and therefore insufficient stability (e.g., ~40 hours at 20 mA cm^−2^) (*1*).

Obviously, catalysts with either AEM or LOM present a trade-off in activity and stability. It is, therefore, crucial to delicately control the reaction pathway between AEM and LOM to realize optimization between activity and stability or ideally achieve dual activation and stabilization of AEM and LOM toward the next generation of highly efficient OER catalysts (*9*). High-entropy alloy (HEA) catalysts are an emerging material platform that has received extensive attention due to their unique structural features, including the high-entropy effect, lattice distortion, slow diffusion, and the “cocktail effect” (*10*–*13*). These properties endow HEAs with simultaneously outstanding structural stability and active site tunability. Intuitively, by rational composition design, the multielement synergy can simultaneously activate both AEM and LOM, while the high entropy configuration can largely improve stability (*1*, *14*). Yet, the preparation of HEA typically requires high-temperature alloying, while the uncontrolled pyrometallurgy can easily lead to particle sintering, phase separation (such as MoC formation, Δ*G*_carburization_ = −94.2 kJ/mol), and severe volatilization (such as Zn, >10-Pa vapor pressure at 1200°C), making these desirable HEA catalysts (dual activation and stabilization) remain unexplored or underdeveloped (*15*–*17*).

**Fig. 1.**
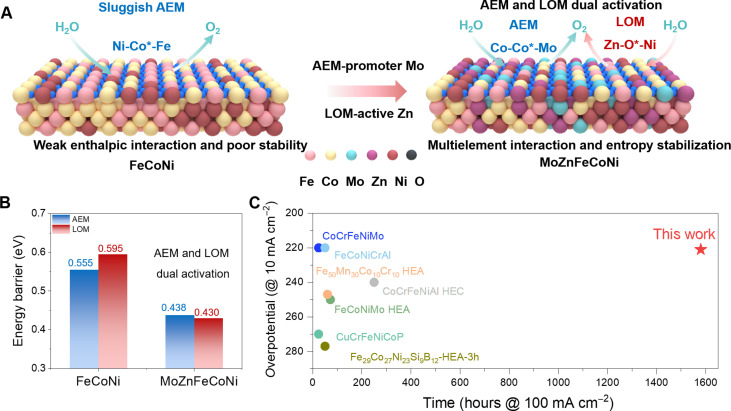
The design of MoZn-based HEA for dual activation and stabilization in OER. (**A**) Schematic of FeCoNi showing sluggish AEM and MoZn-based HEA with dual activation of AEM and LOM. (**B**) Calculated limiting energy barriers in different OER mechanisms, proving the dual activation in MoZnFeCoNi. (**C**) Comparative analysis of catalyst activity and stability following recent literature.

Here, we report the rational incorporation of AEM-promoter Mo and LOM-active Zn into FeCoNi alloy, achieving MoZn-based HEA (MoZnFeCoNi) catalysts that show superior OER activity and particularly long-term stability, owing to the dual activation and stabilization of AEM and LOM (Fig. 1A). Specifically, by density functional theory (DFT) calculation, compared to FeCoNi showing higher reaction energy barriers (AEM: 0.555 eV and LOM: 0.595 eV), MoZn-based HEA exhibits much lower energy barriers for both AEM and LOM (AEM: 0.438 eV and LOM: 0.430 eV; Fig. 1B), where Co-Co^†^-Mo sites largely facilitate AEM to enable rapid deprotonation, while Zn-O^†^-Ni sites follow LOM and can trigger efficient O─O coupling, thus proving dual activation. The multielement interaction (e.g., MoNi and ZnNi pairs), entropy stabilization, and the 3D carbon network are critical to stabilizing the whole structure for long durable OER. As a result, the MoZn-based HEA shows an ultralow overpotential of 221 mV at 10 mA cm^−2^ and outstanding long-term stability with negligible activity loss at 100 mA cm^−2^ for >1500 hours, considerably superior to its counterpart without Mo/Zn and other HEA anodes (Fig. 1C). This work thus offers an approach to enable dual activation and stabilization of OER mechanisms in HEA catalysts and serves as a feasible strategy to concurrently enhance OER activity and stability.

## RESULTS

**Fig. 2.**
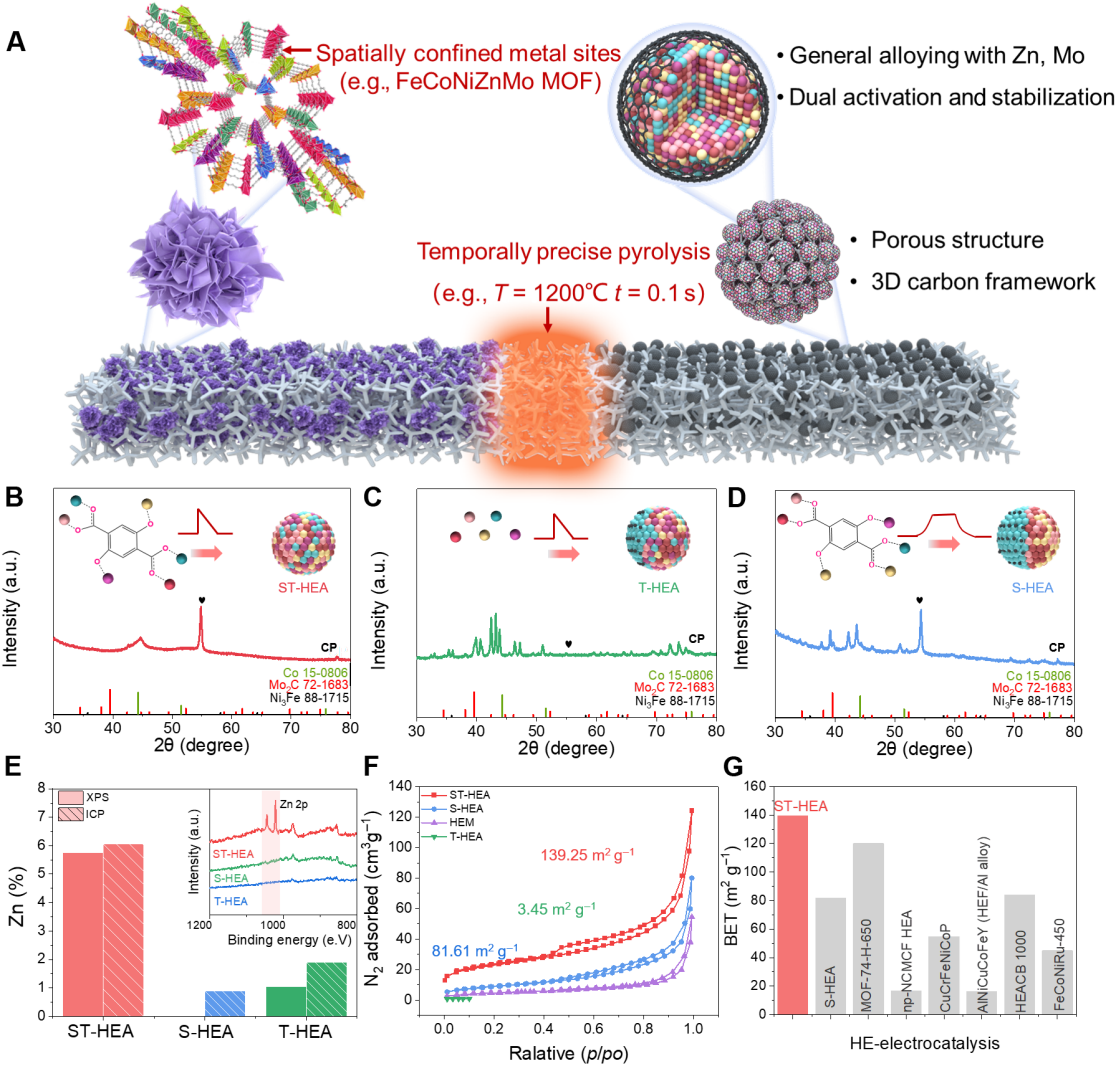
Synthesis and structural characterization of MoZn-based HEA and control samples. (**A**) Considering easily reactive Mo and Zn, the spatial-temporal confined synthesis offers advantages in the synthesis of MoZn-based HEA catalysts. (**B** to **D**) XRD analysis and (**E**) the Zn content measured by x-ray photoelectron spectroscopy (XPS) and inductively coupled plasma optical emission spectrometer (ICP-OES) of ST-HEA, T-HEA, and S-HEA. (**F**) N_2_ adsorption-desorption isotherms of different samples and (**G**) Brunauer, Emmett, and Teller (BET) values for high-entropy catalysts recently reported in the literature. a.u., arbitrary units.

Although Mo and Zn are beneficial for OER, they are usually hard for single phase alloys because of either the oxidation or carburization reaction in Mo and the easy volatility of Zn at high temperatures. For example, the vapor pressure of Zn is >10 Pa at 1200°C, indicating serious volatility. This is in sharp contrast to typical FeCoNi, with a vapor pressure of 10^−8^ Pa. Therefore, we specifically designed a spatial-temporal confined strategy by combining spatially confined precursors [e.g., MoZnFeCoNi metal nodes in metal-organic frameworks (MOFs)] and temporally precise thermal shock pyrolysis (e.g., 1200°C, 0.1 s) to synthesize MoZn-based HEA (Fig. 2A) (*18*). The initial high-entropy MOF structure benefits facile alloying as these metal elements are already highly mixed at the atomic scale in the precursor. Meanwhile, a rapid thermal shock process is used so that the high temperature is essential to trigger uniform alloying while the short duration avoids potential Mo reaction and Zn evaporation. The ligand coordination largely avoids rapid Zn evaporation during the alloying process and eventually forms a hierarchically porous structure with a three-dimensional (3D) interconnected carbon framework, which is deemed beneficial for active site exposure, mass transport, and catalyst stability.

In detail, first, the 2,5-dihydroxyterephthalic acid (H_4_DOBDC) ligand with various metal ions including Ni^2+^, Fe^3+^, Co^2+^, MoO_4_^−^, and Zn^2+^ were assembled as the building blocks of the MoZnFeCoNi high-entropy MOF, where the metal sites are spatially confined in the MOF structure. Then, carbothermal shock (CTS) pyrolysis (i.e., 1200°C for 0.1 s) could trigger instant MOF decomposition and carbonization, while the short duration is critical to avoid severe metal evaporation (e.g., Zn) and carbide formation (e.g., Mo_2_C). Meanwhile, the rapid pyrolysis inherits the multiscale porous structure of the MOF and results in a unique hierarchically porous architecture interconnected by a 3D carbon framework, forming MoZnFeCoNi HEA by spatiotemporal confined synthesis (i.e, ST-HEA). Control samples were also prepared, including (i) precursor metal ions without ligand or MOF formation followed by thermal shock pyrolysis (i.e., only temporal confinement, named T-HEA) and (ii) MOF precursor pyrolyzed by furnace heating at 1000°C for 3 hours under Ar atmosphere (i.e., only spatial confinement, named S-HEA).

X-ray diffraction (XRD) analysis was conducted on catalysts synthesized using various strategies (Fig. 2, B to D). As shown in Fig. 2B, the XRD patterns of ST-HEA illustrate a clear face-centered cubic (FCC) structure with distinct diffraction peaks attributed to (111), (200), and (220) planes. Especially, compared with the diffraction peaks of Fe-based alloy and Ni-based alloy, the position of the diffraction peaks of the ST-HEA is shifted. Furthermore, the other peaks can be assigned to commercial carbon paper (CP) substrate. This proves that these elements were successfully introduced into the nanocatalysts to form a single-phase HEA structure. However, without the proper spatial or temporal confinement, T-HEA and S-HEA lead to obvious second phases, as indicated in the XRD of carbides and other phases (Fig. 2, C and D). To further investigate the content of volatile Zn, x-ray photoelectron spectroscopy (XPS) was performed. The content ratio of the Zn element in ST-HEA, T-HEA, and S-HEA are determined as 5.75, 1.05, and 0%, respectively (Fig. 2E and table S1). In addition, the bulk composition is also confirmed by the inductively coupled plasma optical emission spectrometer (ICP-OES). It can be seen that the volatile element Zn can be retained to the greatest extent by our spatial-temporal confined synthesis, which is consistent with the XPS results (Fig. 2E and table S1). These results further prove the successful synthesis of MoZn-based HEA by spatial-temporal confinement that avoids element evaporation and easy side reactions.

Furthermore, the ST-HEA catalysts inherit the high specific surface area of MOF, which is revealed by the N_2_ isothermal adsorption-desorption isotherm. As shown in Fig. 2F, the surface area of the ST-HEA was analyzed by the BET (Brunauer, Emmett, and Teller), showing an exceptional BET surface area of 139.25 m^2^/g, higher than T-HEA (3.45 m^2^/g) and S-HEA (81.61m^2^/g). The BET surface of ST-HEA is outstanding among alloy particles derived from conventional pyrolysis (86.21 m^2^/g) and various reported high-entropy alloy catalysts (Fig. 2G and table S2), benefited by the inherited 3D porous structure from the rapid pyrolysis of MOF (*19*).

**Fig. 3.**
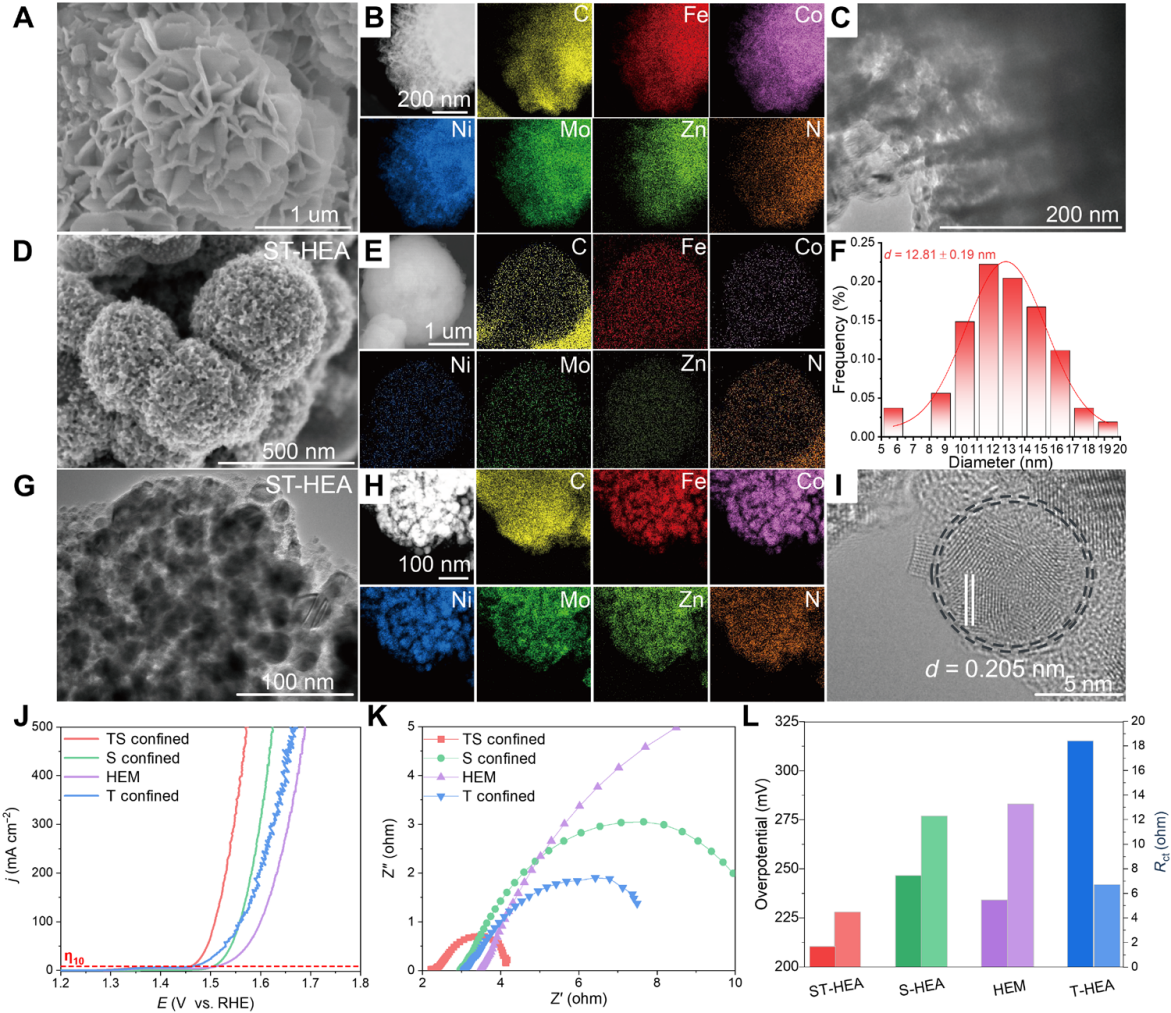
Detailed characterization of MoZn-based HEA. (**A**) SEM image of high-entropy MOF and (**B**) EDS mapping of Fe, Co, Ni, Mo, Zn, C, and N elements with uniform dispersions. (**C**) TEM images of high-entropy MOF. (**D**) SEM images of ST-HEA. (**E**) EDS mapping images of Fe, Co, Ni, Mo, Zn, C, and N elements with uniform dispersions. (**F**) Particle size distribution of ST-HEA. (**G**) TEM of a single multilayered ST-HEA. (**H**) EDS mapping of Fe, Co, Ni, Mo, Zn, C, and N elements with uniform dispersions. (**I**) HRTEM images of HEA present the HEAs encapsulated by the carbon shell layer. (**J**) OER polarization curves, (**K**) EIS Nyquist plots, and (**L**) the comparison of the overpotential at 10 mA cm^−2^ and charge transfer resistance (*R*_ct_) of ST-HEA, S-HEA, high-entropy MOF, and T-HEA catalysts.

The morphology and elemental distribution of MoZn-based HEA were examined by scanning electron microscopy (SEM), transmission electron microscopy (TEM), and energy-dispersive x-ray spectroscopy (EDS). As shown in Fig. 3A, the high-entropy MOF precursor exhibits a flower-like structure composed of staggered and overlapping sheets with a large surface area, which serves as the basis for the formation of the hierarchical structure in HEA. More specifically, elemental EDS mapping of the HEM confirms that six elements, C (yellow), Fe (red), Co (purple), Ni (blue), Mo (green), and Zn (light green) are homogeneously distributed over the selected particle area, as depicted in Fig. 3, B and C. After the MOF precursor was converted to ST-HEA, the SEM result in Fig. 3D showed the shape of a highly porous sphere self-assembled by numerous nanoparticles connected by the carbon framework (derived from the pyrolyzed ligand in the MOF precursor). EDS mapping of ST-HEA demonstrates that the five elements are homogeneously dispersed without element segregation after pyrolysis at high temperatures (Fig. 3E). Also, the real composition of MOF precursor and ST-HEA are shown in table S3.

A more detailed microstructure of the ST-HEA was analyzed by TEM in Fig. 3F, where ST-HEA displays a uniform nanoparticle morphology with a size of 12.81 ± 0.19 nm, interconnected by the carbon framework. These nanoparticles also demonstrate a uniform element distribution to indicate HEA formation throughout the structure (Fig. 3, G and H). In the high-resolution image (Fig. 3I and fig. S1), the lattice fringes were identified as 0.205 nm, ascribed to the (111) crystal face of HEA. Notably, a thin carbon shell (derived from the MOF template) covers the alloy particles seamlessly. Such a highly porous structure enables full exposure of active sites and electrolyte penetration for improved catalytic performance. The interconnected carbon framework is also important to protect the HEA catalysts from easy dissolution during long OER catalysis.

To emphasize the significance of the spatial-temporal confined synthesis, the electrocatalytic OER activity of ST-HEA and other control catalysts were tested in a three-electrode system in N_2_-saturated 1.0 M KOH solution at room temperature (Fig. 3, J to L). The ST-HEA presents the earliest current response compared with T-HEA and S-HEA. The ranking (Fig. 3J) of overpotential (η) at 10 mA cm^−2^ is as follows: ST-HEA (221 mV) < T-HEA (242 mV) < S-HEA (276 mV). Notably, despite the surface carbon coating, our ST-HEA shows better activity than T-HEA (without carbon), suggesting better dispersion by the carbon coating and nonpoisoning of the active sites (fig. S2). These results suggest that our strategy could contribute to higher activity by enabling MoZn-HEA. Electrochemical impedance spectroscopy (EIS) analysis was performed to probe the charge transfer phenomena at the catalyst-electrolyte interface. In Fig. 3K, Nyquist plots consist of two semicircles, the former signifies the electron transfer from catalyst to the working electrode, while the latter reflects the electron-mediated electrochemical oxidation of water molecules. The charge transfer resistance (*R*_ct_) of ST-HEA is 2.5 ohm, far lower than control materials, thus demonstrating a much faster charge transfer during the OER process on ST-HEA, which can be attributed to the hierarchically porous structure and graphitized carbon shells (Fig. 3L). This result further illustrates that the porosity of the ST-HEA is expected to supply more catalytic active sites, leading to the enhancement of their charge and mass transport.

**Fig. 4.**
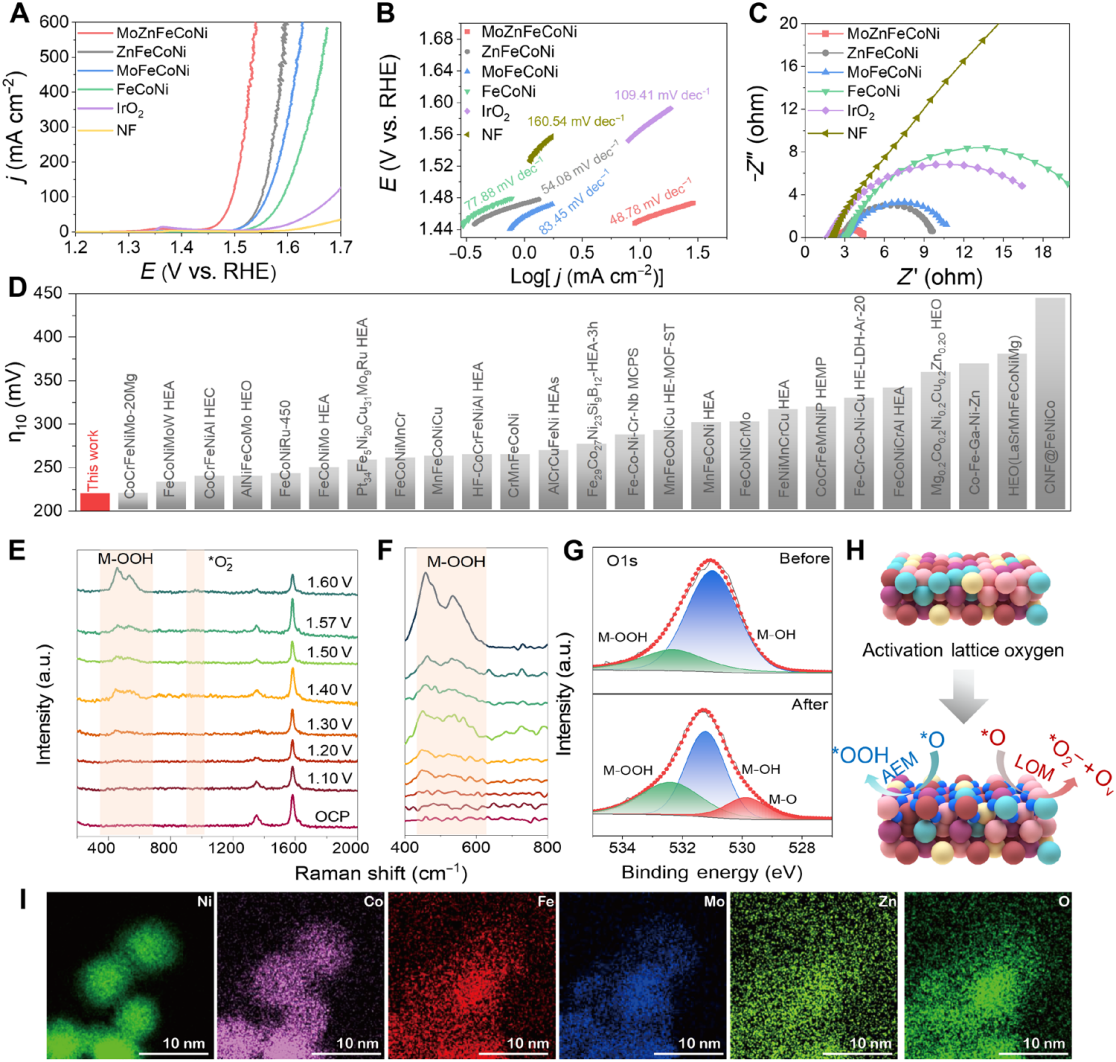
The electrocatalytic properties of the prepared catalysts and commercial catalysts. (**A**) IR-corrected polarization curves at 10 mA cm^−2^ and (**B**) Tafel plots of the MoZnFeCoNi, ZnFeCoNi, MoFeCoNi, and FeCoNi catalysts in 1.0 M KOH solution at room temperature. (**C**) Nyquist plots of the MoZnFeCoNi, IrO_2,_ and NF EIS. (**D**) Comparison of overpotentials at 10 mA cm^−2^ for MoZnFeCoNi catalyst with recently reported high-entropy OER electrocatalysts in 1.0 M KOH. (**E** and **F**) In situ Raman spectra collected on MoZnFeCoNi for the OER in 1 M KOH. (**G**) XPS analysis of the catalyst before and after OER activation of O 1 s. (**H**) Schematic representation of the surface reconstruction and dual activation of OER mechanisms of MoZnFeCoNi composite after OER activation. (**I**) Corresponding EDX mapping images of MoZnFeCoNi.

To explore the role of incorporating Mo and Zn in HEA on the OER activity, the performances of MoZnFeCoNi, FeCoNi, ZnFeCoNi, MoFeCoNi (all synthesized by the same method), commercial IrO_2_ catalysts, and nickel foam (NF) were studied for OER under the same conditions (Fig. 4A). According to the polarization curves shown in Fig. 4A and fig. S3, the MoZnFeCoNi catalyst exhibits the lowest overpotential of 221 mV at a current density of 10 mA cm^−2^ compared to that of the FeCoNi (300 mV), ZnFeCoNi (277 mV), MoFeCoNi (278 mV), commercial IrO_2_ (337 mV), and NF (391 mV). Impressively, the obtained MoZnFeCoNi catalyst achieves an ultralow overpotential of 341 mV at a high current density of 500 mA cm^−2^, indicating great potential for practical application. The Tafel slope of the MoZnFeCoNi catalyst (48.78 mV dec^−1^) is also lower than that of IrO_2_ (109.41 mV dec^−1^) (Fig. 4B), confirming a faster OER kinetics on the MoZnFeCoNi catalyst.

It is known that the electrochemically active surface area (ECSA) of the electrocatalyst is proportional to the electrochemical double-layer capacitance (*C*_dl_), which can be evaluated by measuring the scan rate–dependent cyclic voltammetry (CV) in the non-Faraday region (figs. S4 and S5). As shown in fig. S6, the measured *C*_dl_ value of the MoZnFeCoNi catalyst is 11.84 mF cm^−2^, which is a bit higher than that of IrO_2_ (6.04 mF cm^−2^) catalysts. We then compared the ECSA-normalized OER activities of the catalysts, which still follow the order of MoZnFeCoNi > ZnFeCoNi > MoFeCoNi > FeCoNi (fig. S7). These results indicated that the intrinsic OER activity of MoZnFeCoNi is still superior to that of other control samples, suggesting improved OER kinetics from the catalysts consideration.

To further illustrate the electrode reaction kinetics, EIS was performed for the above samples. As shown in Fig. 4C, the Nyquist plots reveal that MoZnFeCoNi has the lowest *R*_ct_ of about 2.5 ohm at the overpotential of 300 mV in 1.0 M KOH, which is much smaller than that of the commercial IrO_2_ (18.3 ohm), demonstrating the highest electronic conductivity and much faster charge transfer during the electrochemical OER process. Given the low overpotential at 10 mA cm^−2^, the OER activity of MoZnFeCoNi also surpasses many recently reported OER high-entropy electrocatalysts, as summarized in Fig. 4D and table S4 (*3*, *20*–*36*). To further optimize the OER performances, different MoZn ratios in MoZn-based HEA were prepared. As shown in fig. S8, as Zn increases, the OER activity first increases and then decreases. This result shows that by tuning the MoZn ratios, the OER properties can accordingly change and the proper MoZn ratio leads to optimal OER activity. This may be balanced by increasing Zn to trigger efficient LOM yet without compromising the structural integrity and exposure of AEM active sites. Moreover, excessive Zn or LOM can cause structural destabilization and poor activity (*1*). These results confirmed the dual activation in MoZnFeCoNi HEA, which facilitates both AEM and LOM to achieve a balance of activity and stability.

To identify the actual OER active sites of MoZnFeCoNi, in-situ and operando Raman spectra were performed using a customized electrochemical cell, as shown in Fig. 4E. At 0 to 1.20 V versus a reversible hydrogen electrode (RHE), MoZnFeCoNi did not show spectral peaks, indicating that the catalyst had not been oxidized at this voltage. With an increase in the applied voltage from 1.2 to 1.4 V versus RHE or the residence time of the catalyst in the electrolyte, as a general trend, a pair of bands at 473.0 and 552.4 cm^−1^ appeared on the as-prepared catalysts assigned to M-O vibrations in M-OOH (M = Fe, Co, Ni, Mo, Zn) (Fig. 4, E and F), indicating the structural reconstruction of catalyst during the OER process. In addition, in situ Raman can also observe changes in reaction intermediates during the OER process (*37*, *38*). The other three peaks at 808, 914, and 980 cm^−1^ are all characteristic peaks of oxygen-containing intermediates (O*, OH*, and OOH*). Moreover, the Raman peak at 1063 cm^−1^ is regarded as *O_2_^−^ gradually becoming stronger and sharper with the potential increasing, which may be an intermediate product of activated lattice oxygen (Fig. 4, E and F). The presence of oxygen-containing intermediates and *O_2_^−^ species indicates AEM and LOM dual activation in the MoZnFeCoNi OER process.

High-resolution XPS was used to further explore the structure of the catalyst after activation. Compared to ST-HEA before activation, the ratios of Fe^0^/Fe^2+^ (about 1.501), Co^0^/Co^2+^ (about 1.204), Ni^0^/Ni^2+^ (about 1.56), Mo^0^/Mo^2+^ (about 1.59), and Zn^0^/Zn^2+^ (about 1.510) for ST-HEA after the activation test are slightly decreased (fig. S9). Meanwhile, Fig. 4G shows the three oxygen peaks at 530.2, 531.7, and 532.8 eV that represent lattice oxygen (M-O), defective oxygen (M-OH), and adsorbed oxygen species (M-OOH), respectively. The high-resolution XPS survey spectrum after the activity test for the MoZnFeCoNi catalyst reveals the presence of M-O (23.58 atomic %), suggesting that the metals are gradually oxidized to generate MOOH, which is considered to be the true catalytic species of OER (table S5) (*39*, *40*). This result is consistent with the results of Raman spectroscopy (Fig. 4, G and H). The content of MoZn-based HEA after activation exhibits slight dissolution due to metal reconstruction to corresponding oxides or hydroxides (table S6). The activated HEA was characterized by corresponding high-resolution TEM (HRTEM) and EDX mapping. The superposition of the inverse fast Fourier transform images, plotted using the blue and red reflection points corresponding to MOOH and HEA matrix in fig. S10, confirms that the actual OER active sites of MoZnFeCoNi are MOOH (Fig. 4I).

**Fig. 5.**
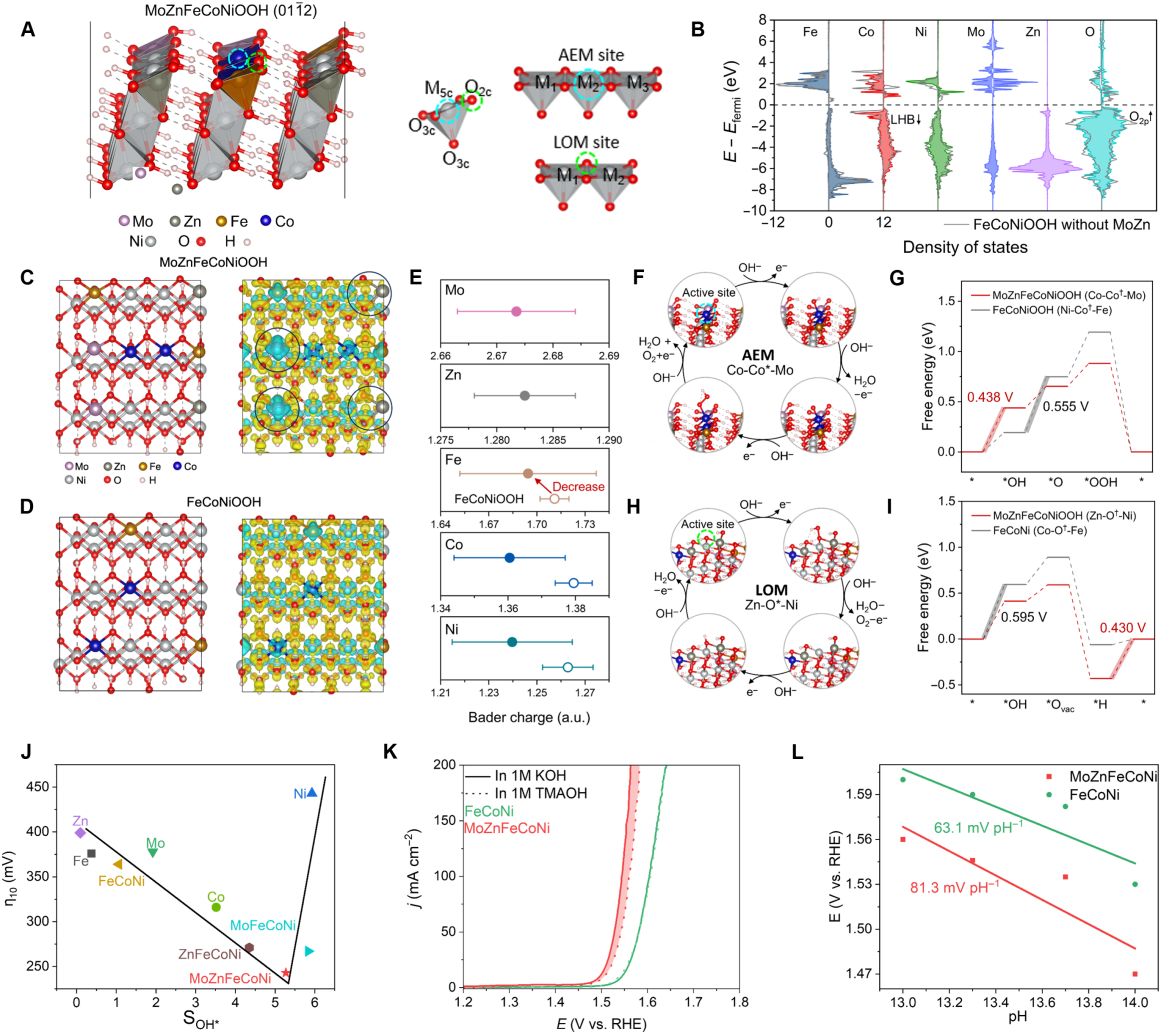
DFT calculation and mechanism analysis in the OER process. (**A**) Model of the MoZnFeCoNiOOH (01$\bar{1}$2) facet for DFT calculations. The blue and green circles denote the active sites for AEM and LOM pathways. The coordination of metal and oxygen atoms is marked, where 5c, 3c, and 2c represent five-, three-, and twofold coordination. The M_1_-M_2_*-M_3_ and M_1_-O*-M_2_ are the arrangements that are critical to AEM and LOM performance. (**B**) PDOS of surface Fe, Co, Ni, Mo, Zn, and O atoms in the MoZnFeCoNiOOH model, where the results of FeCoNiOOH are presented as gray lines. (**C** and **D**) Slab model and charge density difference of MoZnFeCoNiOOH and FeCoNiOOH. Yellow and blue colors represent electron accumulation and depletion, respectively, with an iso-surface value of 0.02 eÅ^−3^. (**E**) Statistical analysis of Bader charges at sites on the outer surface of MoZnFeCoNiOOH (solid dots) and FeCoNiOOH (open dots). The dot and error bar show the average and SD of the Bader charges. (**F** and **G**) AEM pathway and the corresponding Gibbs free energy diagram of OER steps of the Co-Co*-Mo site. The free energies are obtained at 1.23 V versus RHE. The free energy diagram of FeCoNiOOH is presented for comparison. (**H** and **I**) LOM pathway and the corresponding Gibbs free energy diagram of OER steps of the Zn-O*-Ni site. (**J**) Relationship between the overpotential in the MOR and the current difference leading to the filled area in catalysts. (**K**) LSV curves of FeCoNi and MoZnFeCoNi in 1.0 M KOH and 1.0 M TMAOH, respectively. (**L**) Relationship between the logarithm of the current density of MoZnFeCoNi and FeCoNi at the potential of 1.5 V versus RHE and pH.

DFT calculations are conducted to clarify the OER mechanisms of MoZnFeCoNi. Generally, OER electrocatalysts reconstruct into (oxy)hydroxides on their surface, with MOOH functioning as the actual catalytic material (*1*, *41*). Consequently, MoZnFeCoNiOOH is used as a representative model. We construct a model featuring an interior of β-NiOOH with surface-doped with MoZnFeCo (as shown in Fig. 5A) based on the high content of Ni in the alloy (Fig. 2I), and select the widely studied lateral (01$\bar{1}$2) facets for investigation (*42*). Spin-polarized DFT + *U* calculations with Grimme’s D3 correction are performed (Materials and Methods). Both AEM and LOM pathways are considered to identify the active sites. As depicted in Fig. 5A, the five-coordinated unsaturated metal sites (M_5c_) act as active sites for AEM, while the two-coordinated oxygen atoms (O_2c_) can engage in the OER via the LOM pathway. In the AEM pathway, not only the center metal atom (denoted as M_2_) but also adjacent metal atoms M_1_ and M_3_ influence the reaction. This M_1_-M_2_^†^-M_3_ arrangement is crucial for the AEM analysis, where the active site is decorated with ^†^. For the LOM pathway, the M_1_-O^†^-M_2_ arrangement is critical, with the metals adjacent to the O_2c_ atom playing substantial roles.

Figure 5B shows the partial density of states (PDOS) of the surface atoms in the MoZnFeCoNiOOH model, where the results of FeCoNiOOH without MoZn are also presented. The strong *d-d* Coulomb interaction splits the *d* orbitals into a filled lower Hubbard band (LHB) and an empty upper Hubbard band (UHB) (*1*). The centers of LHB and UHB of the surface as well as each element type are calculated and presented in table S7 (Materials and Methods). We find that Co atoms have the highest LHB center, and the states near *E*_fermi_ are mainly contributed by Co atoms (Fig. 5B). This is in accordance with our results showing that Co sites demonstrate the lowest AEM overpotential. After adding Mo and Zn, the overall LHB center downshifts from −4.342 to −4.794 eV. Meanwhile, the O-2p band upshifts from −2.480 eV of the FeCoNiOOH to −2.210 eV of the MoZnFeCoNiOOH (Fig. 5B). The downward shift of the LHB center and the upward shift of the O-2p center in MoZnFeCoNiOOH lead to a reduced overlap between the metal *d* orbitals and O-2p orbitals, which weakens the metal-oxygen bond and can trigger the LOM mechanism (*1*, *8*, *43*). Fig. 5 (C and D) presents a comparison of the charge density difference of MoZnFeCoNiOOH and FeCoNiOOH, and Fig. 5E shows a statistical analysis of Bader charges on the surface metal sites. We observe notable charge depletion around the Mo atoms (Fig. 5C), and the Bader charges are ~2.67, reflecting a high-valence state. Zn adopts a low-valence Zn^2+^ state with a completely filled 3d electron shell. This results in electron localization around the O atom along the Zn─O bond and weakens the bond strength (Fig. 5C). Compared to that of FeCoNiOOH, the average Bader charges of Fe, Co, and Ni sites overall decrease (Fig. 5D), and their SDs notably increase. This change is in accord with previous reports when adding high-valence elements into oxyhydroxide (*44*). This redistribution modulates the electronic structures of both the metal and O sites (Fig. 5B), which is crucial for optimal OER performances.

Next, the OER performances of active sites in MoZnFeCoNiOOH and FeCoNiOOH are examined by DFT calculations, with both AEM and LOM pathways considered (Materials and Methods). For the M_1_-M_2_^†^-M_3_ arrangement in the AEM scenario, Co atoms exhibit the highest LHB center, and the states near the Fermi level are predominantly contributed by Co 3d electrons (Fig. 5B), signifying Co as the primary active site. Hence, we focus on the Co atom as the active center (M_2_ = Co) and explore the impact of neighboring atoms (M_1_, M_3_ = Mo, Zn, Fe, Co, and Ni) on its OER performance. We investigated $C_{5}^{2}$ = 10 scenarios, with M_1_ and M_3_ involving a pair from the five elements (pairs where M_1_ and M_3_ that are identical are not considered here), which can be categorized into two groups: (i) pre-existing combinations such as Fe-Co^†^-Ni, Fe-Co^†^-Co, and Co–Co^†^-Ni, which are also present in FeCoNiOOH and (ii) new combinations such as Mo-Co^†^-Fe, Mo-Co^†^-Co, Mo-Co^†^-Ni, Mo-Co^†^-Zn, Zn-Co^†^-Fe, Zn-Co^†^-Co, and Zn-Co^†^-Ni, arising from the addition of Mo and Zn. The slab models, intermediate adsorption configurations (*O, *OH, and *OOH) and the Gibbs free energies diagrams for each combination are presented in fig. S11. When the Co site is adjacent to Co and Mo and forming a Co-Co^†^-Mo site, the overpotential can be lowered to 0.438 V (Fig. 5, F and G). This is caused by the slight increase in the energy cost of adsorbing *OH, which becomes the potential-determining step (PDS) but optimizes the overall overpotential by facilitating the conversion from *OH to *O (Fig. 5G). In the FeCoNiOOH case, the Ni-Co^†^-Fe site shows the lowest overpotential (0.555 V), and the PDS is the deprotonation of *OH (Fig. 5G).

As to the LOM pathway, the O atoms between different metals serve as the active centers, denoted as M_1_-O^†^-M_2_. We also investigated 10 scenarios to explore the neighboring effects: (i) pre-existing combinations such as Fe-O^†^-Ni, Fe-O^†^-Co, and Co-O^†^-Ni, common in FeCoNiOOH and (ii) new combinations such as Mo-O^†^-Fe, Mo-O^†^-Co, Mo-O^†^-Ni, Mo-O^†^-Zn, Zn-Co^†^-Fe, Zn-Co^†^-Co, and Zn-Co^†^-Ni, stemming from the inclusion of Mo and Zn. The single-site LOM pathway that involves the formation and refilling of lattice oxygen vacancy is considered (Materials and Methods). The detailed results for these M_1_-O^†^-M_2_ combinations are presented in fig. S12. The new inclusion of Zn introduces the Zn-Co^†^-Ni and Zn-Co^†^-Co sites, which show optimal overpotentials of 0.430 V and 0.538 V, respectively (Fig. 5, H and I, and fig. S12). These improvements are attributed to the upshift of the O-2p band center and the weakening of Zn─O bonds, which enhances the activity of lattice oxygen. For the FeCoNiOOH, the PDS is the first step namely the attack of the hydroxyl group, yielding an overpotential of 0.595 V (Fig. 5I). This overpotential is higher than that of the AEM case (0.555 V), indicating that LOM is not favored in FeCoNiOOH. In the case of MoZnFeCoNiOOH, the overpotentials of AEM and LOM, i.e., 0.438 V and 0.430 V, are quite comparable, suggesting a large possibility of dual activation of these pathways. This synergy uses both metal and oxygen atoms as active sites, notably increasing the OER performance. We also would like to note that our investigated sites are still restricted, and the reported dependency of OER performance on elemental combinations represents a general trend. The catalytic performance can also be influenced by the intricate bond lengths, angles, and potentially the local environments within a cutoff distance of up to 5 to 10 Å. In the future, we plan to construct a larger dataset and use a machine learning–assisted approach to build a more comprehensive relationship. Despite this, our findings still provide useful insights into the general trend of chemical impact on OER mechanisms.

To experimentally confirm the dual mechanism activation, the methanol oxidation reaction (MOR) and the tetramethylammonium cation (TMA^+^) molecular probe were used to investigate the oxygen transfer path under AEM or LOM dominance. Three oxygen-containing intermediates *OH, *O, and *OOH (* refers to the surface ctive site) are generated during the four-electron transfer process of OER during AEM (*2*). The nucleophilic reagent methanol can easily adsorb the electrophilic reagent *OH in the process of OER, which forms a competitive relationship with the adsorption of *OH by OER. Therefore, the MOR is considered as an electronic probe assaying the adsorption of OER intermediates, that is, the increase of current density in MOR is positively correlated with the coverage of intermediate *OH.

We then probed the MOR and *OH adsorption for MoZnFeCoNi, individual elements, and other catalysts by CV (figs. S13 and S14). The filled areas between the LSV curves with and without methanol were calculated to reflect the extent of surface coverage of *OH (S_MoZnFeCoNi_ = 5.27, S_FeCoNi_ = 1.06, S_MoFeCoNi_ = 5.83, S_ZnFeCoNi_ = 4.35, S_Fe_ = 0.37, S_Co_ = 3.51, S_Ni_ = 5.92, and S_Mo_ = 1.91, and S_Zn_ = 0.09). With an increase in the adsorption of the reaction intermediate *OH, the OER electrocatalytic activity of these catalysts exhibits a volcano-like trend (Fig. 5J), where the MoZnFeCoNi shows the highest OER activity and moderate *OH adsorption. According to Sabatier’s principle, excessively strong or weak adsorption is not conducive to efficient catalysis, and the moderate adsorption in MoZnFeCoNi proves its optimal OER reactivity through the activated AEM process.

On the other hand, in the LOM route, due to direct O─O coupling bypasses, the correlated intermediate adsorption in the AEM, superperoxo-like (O_2_^−^), and peroxo-like (O_2_^2−^) species was generated. On the basis of this phenomenon, the TMA^+^ can be introduced as a chemical probe to trace these negatively charged oxygen-containing species by binding strongly to the negative intermediates. This can be revealed by the fact that the OER activity of MoZnFeCoNi sharply decreased after adding the TMA^+^ species to the electrolyte, while the OER activities of FeCoNi remained nearly unchanged after adding TMA^+^ species (Fig. 5K and fig. S15). This result confirms that the introduction of Zn will cause the catalyst to produce a large amount of oxygen-containing intermediates during OER, thus proving the effective triggering of the LOM, while FeCoNi shows minimum change and does not trigger LOM. Meanwhile, because LOM is a nonconcerted proton-electron transfer pathway and exhibits strong pH-dependent OER activity, we also investigated the pH-dependent OER activity of the MoZnFeCoNi, FeCoNi, ZnFeCoNi, and MoFeCoNi. As displayed in Fig. 5L, the OER activity of MoZnFeCoNi was highly dependent on the pH of the electrolyte, whereas this pH dependence was very weak on catalysts without Mo and Zn, further suggesting LOM in MoZnFeCoNi. Therefore, the above experimental results (methanol probe, TMA probe, and pH dependence) confirm the dual activation of AEM and LOM in MoZnFeCoNi HEA.

**Fig. 6.**
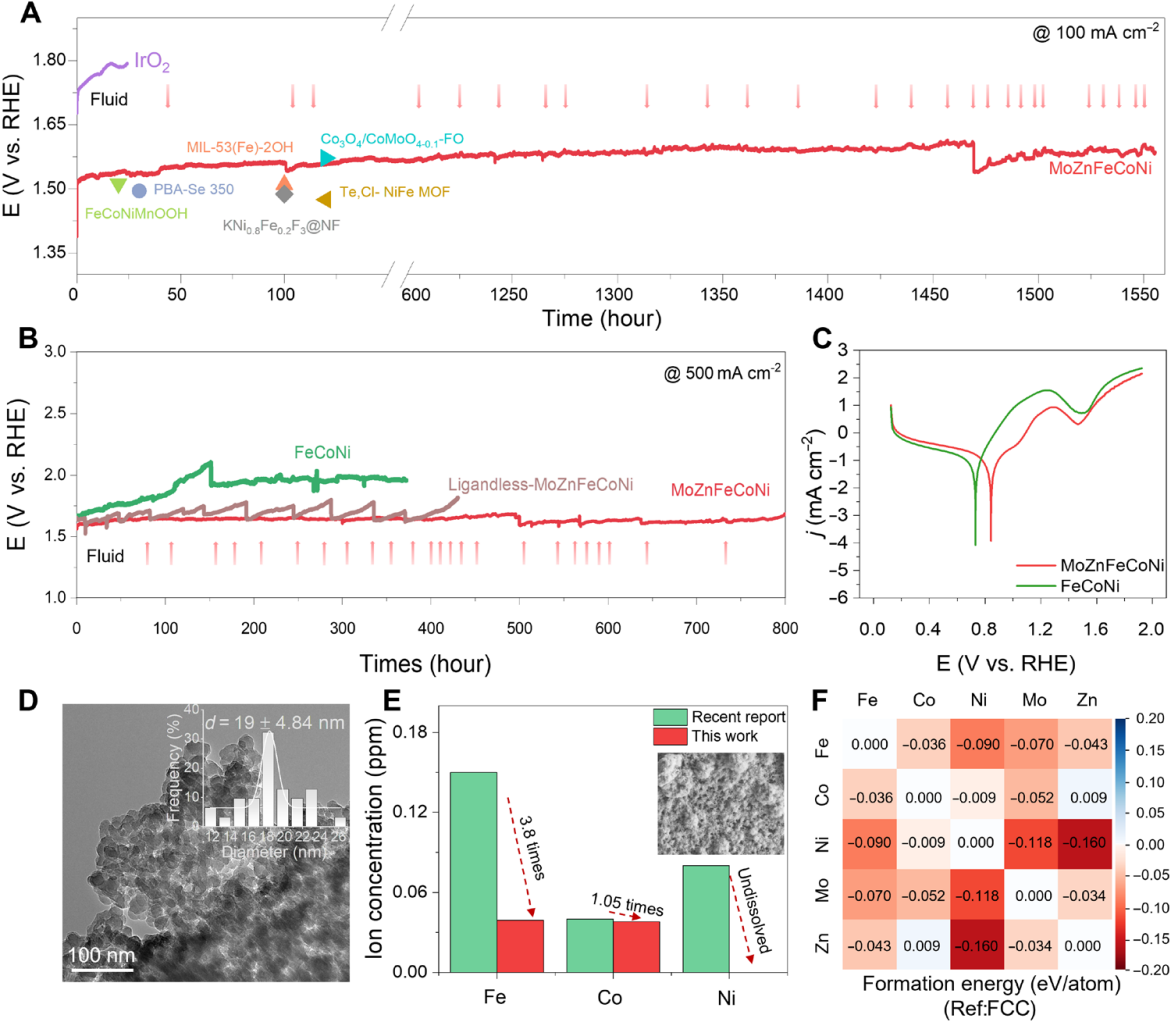
Electrocatalytic OER stability of the MoZnFeCoNi. (**A**) Catalytic stability by chronopotentiometry measurement in 1.0 M KOH at 100 mA cm^−2^ and (**B**) at 500 mA cm^−2^. (**C**) Corrosion potential analysis of the catalysts. (**D**) TEM images of MoZnFeCoNi after stability test at 500 mA cm^−2^ (the inset is the particle size distribution of single particles in hierarchical MoZnFeCoNi). (**E**) Corresponding ion concentrations in electrolyte after stability test at a constant current density of 100 mA cm^−2^. (Inset SEM is the carbon network of the MoZnFeCoNi after strong acid etching.) (**F**) Formation energies between element pairs in MoZnFeCoNi with reference to the FCC structures of constituent elements.

Besides the superior catalytic activity, long-term operational stability is undoubtedly another important performance parameter that affects the wide application of OER electrocatalysts (*4*). In this work, we assessed the stability of MoZnFeCoNi using a chronopotentiometry (CP) method by applying a voltage that maintains a current density of 100 mA cm^−2^ in a 1.0 M KOH electrolyte. As shown in Fig. 6A, the MoZnFeCoNi demonstrated a sustained catalytic activity for >1500 hours, representing a substantial improvement over commercial IrO_2_ (only lasting for 24 hours). Moreover, the stability of the catalyst at this current density exceeds that of most catalysts reported so far (typically <100 hours) (table S8). The stability of MoZnFeCoNi, ligandless MoZnFeCoNi (i.e., no carbon shell), FeCoNi, and FeCoNiZn (i.e., less intermetallic bonding and entropy effect) were further evaluated by maintaining a current density of 500 mA cm^−2^. From Fig. 6B, the potential of the MoZnFeCoNi is almost unchanged after 800 hours stability tests at 500 mA cm^−2^. In contrast, the apparent activity of ST-FeCoNi starts to deteriorate rapidly after 100 hours, suggesting the importance of MoZn incorporation and the formed HEA structure. Meanwhile, the stability of ligandless MoZnFeCoNi also decreased gradually over time. These results suggest the importance of carbon coating and MoZn incorporation for improved OER stability. Particularly, it seems that multielement interaction and the high-entropy structure (after reconstruction) contribute more to the improved durability.

To verify the practical application potential, the MoZn-HEA and commercial Pt/C electrodes were directly used as the anode and cathode for assembling anion-exchange-membrane water electrolyzer (AEMWE). The polarization curves show that a MoZn-HEA–based AEMWE device only needs 1.91 V to reach an industrial current density of 1000 mA cm^−2^. The stability curve of the MoZn-HEA electrolyzer exhibits a well-maintained cell voltage over 350 oursh at 1000 mA cm^−2^ in 1.0 M KOH at 50°C (fig. S16). The superior durability of MoZn-HEA may be due to the hierarchical carbon shell connecting all catalysts as well as the increased multielement interaction and the high-entropy stabilization. Our findings indicate that the MoZn-HEA has notable potential for commercial applications in AEMWE.

The corrosion resistance of the catalysts was evaluated using potentiodynamic polarization (PDP) curves. The PDP curves (Fig. 6C) describe that MoZnFeCoNi and FeCoNi have similar electrochemical corrosion behavior in 1.0 M KOH, but the corrosion potential of MoZnFeCoNi (0.83 V versus RHE) is higher than that of FeCoNi (0.73 V versus RHE), which indicate that MoZnFeCoNi that has superior anticorrosion ability is less prone to corrosion and degradation in the alkaline environment of the electrolyte, thus enhancing the catalytic stability. We also characterized the catalysts after long-term operation. From TEM images and EDX results in figs. S17 and S18, TEM images of MoZnFeCoNi show that the hierarchical structure keeps well and the Fe, Co, Ni, Mo, Zn, C, N, and O elements are still evenly distributed throughout the whole region of the MoZnFeCoNi catalyst, suggesting its excellent stability. The statistical analysis of the particle size of the catalyst after stability testing showed that the single-particle size of the catalyst was substantially larger (inset of Fig. 6D). This increase in size may be caused by surface oxidation following the electrochemical reconstruction of the catalyst (Fig. 6D and fig. S17).

We analyzed the composition of electrocatalysts after the 1500-hour stability test at 100 mA cm^−2^ by ICP-OES (table S3) and found that the Fe/Co/Ni/Mo MoZn-based HEA showed slight change, while Zn showed a larger solubility. The dissolution of Fe, Co, Ni, Mo, and Zn in MoZnFeCoNi in the electrolyte after the stability test is much lower than FeCoNi and ZnFeCoNi (Fig. 6E and table S9). It is also much superior to the advanced FeCoNi OER catalyst reported in the literature (table S9) (*45*). Notably, although Zn has a higher solubility, if we average it through the life cycle (1500 hours), then it only shows a dissolution rate of 1.27 parts per billion/hour. Namely, the dissolution in our MoZnFeCoNi is small during the whole OER process, thus ensuring OER durability. The suppressed dissolution rate from the MoZnFeCoNi may be due to the carbon shell preventing direct contact between the metal and the electrolyte and the increased multielement interaction and the high-entropy stabilization. First, the catalyst was subjected to a 24-hour etching process at 80°C in 1.0 M HCl, which resulted in complete metal etching and the formation of a highly porous carbon network, as observed in the SEM image of the catalyst after etching (inset of Fig. 6E). This allows us to directly observe such a carbon network in our catalysts, which could effectively protect the metal catalysts from rapid dissolution.

DFT calculations were performed to shed light on how Mo and Zn incorporation boosted the long-term durability of MoZnFeCoNi catalysts for OER. In Fig. 6F, the stability analysis is based on FCC formation energy calculations between pairs of elements. In this analysis, the formation energy is calculated with reference to the FCC phases of the constituent elements despite that FCC is not necessarily the most stable allotrope for each element. We use this calculation setup to directly assess the elemental intermixing and entropic stabilization within the alloy and evaluate the alloy’s resistance to segregation and phase separation, which are critical for the long-term stability of catalysts. Notably, Mo and Zn display negative binding energies with almost all other elements (Fig. 6F), and the degree of negativity follows the order Ni > Fe > Co, signifying that Ni favors the strongest intermixing with Mo and Zn, followed by Fe, and then Co. This sequence aligns well with the elemental dissolution result in Fig. 6E. These results reveal that the multielement interaction and high-entropy stabilization facilitated by Mo and Zn incorporation play an important role in improving the structural and long-term OER stability.

## DISCUSSION

This study proposed that the spatial-temporal confined strategy can construct MoZn-based HEA with a hierarchical porous structure interconnected by a 3D carbon framework, which demonstrates superior and simultaneous enhancement in OER activity and stability. The MoZnFeCoNi achieves an ultralow overpotential of 221 mV at 10 mA cm^−2^ and excellent long-term stability at 100 mA cm^−2^ over >1500 hours. DFT calculation and chemical probe experiment confirmed the dual activation of AEM and LOM in MoZn-based HEA catalysts, where Co-Co^†^-Mo can enable rapid deprotonation (AEM), while Zn-O^†^-Ni can trigger O─O coupling step (LOM). The dual activation can break through the linear scaling limit to facilitate highly efficient OER. The multielement interaction, entropy stabilization, and 3D carbon network are critical to stabilizing the whole structure for long durable OER. This work offers a unique strategy to design and tailor the OER mechanisms and achieves dual activation and stabilization in HEA catalysts. The proposed spatial-temporal confined synthesis is also general for HEAs containing easily volatile and reactive elements.

## MATERIALS AND METHODS

### Materials

Iron acetate tetrahydrate [Fe(CO_2_CH_3_)_2_·4H_2_O], cobalt nitrate hexahydrate [Co(NO_3_)_2_·6H_2_O], nickel nitrate hexahydrate [Ni(NO_3_)_2_·6H_2_O], zinc nitrate [Zn(NO_3_)_2_·6H_2_O], carbon nanotubes, ammonium molybdate tetrahydrate [(NH_4_)_6_Mo_7_O_24_·4H_2_O], *N*,*N*-dimethylformamide (DMF), 2,5-dihydroxyterephthalic acid, carbon nanotube pyromellitic dianhydride (KOH, analytial reagent (AR), 90%), and ethanol (EtOH) were bought from Aladdin Reagent.

All of the above chemicals were of analytical grade and used as received without further purification. Ultrapure deionized (DI) water (18.2 megohm cm^−1^) was used in all experiments. Commercial NF (thickness: 1.7 mm) was purchased from Taiyuan Liyuan Lithium Battery Technology Co. Ltd and cut into small pieces, having a geometric area of 1 cm × 3 cm. NF was first ultrasonicated in 0.5 M H_2_SO_4_ for 1 hour to remove the thin oxide layer formed on the surface, then washed with DI water alternately three times, and let dry at room temperature.

### Synthesis of spatio-temporal FeCoNiMoZn HEA

The synthesis of FeCoNiMoZn MOFs started with the dissolution of 0.25 mmol of Fe(CO_2_CH_3_)_2_·4H_2_O, 0.25 mmol of Co(NO_3_)_2_·6H_2_O, 0.25 mmol of Ni(NO_3_)_2_·6H_2_O, 0.25 mmol of Zn(NO_3_)_2_·4H_2_O, 0.25 mmol of Na_2_MoO_4_, and 0.34 mmol of 2,5-dihydroxyterephthalic acid in the mixture of 22.5 ml of DMF, 1.35 ml of anhydrous ethanol, and 1.35 ml of DI water. Then, the mixture sonicated at least 60 min. Subsequently, the mixture was immediately transferred into a 50-ml autoclave and then heated to 120°C for 24 hours. The synthesized FeCoNiMoZn MOFs precursor was washed with ultrapure DI water, DMF, and EtOH in sequence for several times and dried for 12 hours at 70°C. A large current pulse (20 A, 100 ms) was used for CTS in an Ar atmosphere. The current output mode is adopted and the maximum output voltage is 30 V. The fast heating rate of ~104°C/s is achieved by an instant pulsing current to 10 A in 100 ms (~100 A/s), and other slower heating rates (100° and 1000°C/s) can be achieved by programming the current stepwise at rates of 1 and 10 A/s from 0 to 10 A. Last, a heating rate of 0.1°C/s is obtained by conventional furnace treatment (6°C/min).

### Electrocatalytic measurements

Electrochemical measurements of the as-synthesized samples were performed with a CHI760E electrochemistry workstation (CH Instruments Inc.) using a standard three-electrode electrochemical cell with Pt sheet and Hg/HgO as the counter electrode and the reference electrode, respectively. The catalyst ink for OER tests was prepared as following: 5 mg of catalysts was dispersed into a mixed solution of 300 μl of 0.5% Nafion ethanol solution and 200 μl of DI water and then ultrasonicated for 30 min to obtain a homogeneous solution. A total of 50 μl of the catalysts ink was loaded on a clean NF electrode (0.5 cm^2^) by drop coating to form the electrode and dried slowly at room temperature. The final mass loading of the catalysts on electrode is around 1 mg cm^−2^.

The electrochemical measurements were all performed at room temperature, and the potential was referenced to that of the RHE.For the RHE calibration, the potential difference between Hg/HgO and RHE was measured in 99.999% pure H_2_-saturated 1.0 M KOH aqueous solution. During the measurement, high-purity H_2_ is bubbled into the electrolyte to saturate the electrolyte and fix the reversible hydrogen potential.

Cyclic voltammograms were taken several cycles to bubble away the surface contaminates and at the same time stabilize the catalysts. The polarization curves were obtained by linear sweeping with a scan rate of 1.0 mV s^−1^ in O_2_-saturated 1.0 M KOH aqueous solution. Double layer capacitance (*C*_dl_) was estimated by the cyclic voltammogram cures at various scan rates (10 to 50 mV) in the potential region of 0.1 to 0.2 V versus RHE. All the polarization curves were iR-corrected, except as otherwise noted. EIS is recorded at an applied potential of 1.53 V versus RHE from 100 kHz to 0.1 Hz.

CP responses curve was performed under a high constant current density of 100 mA cm^−2^ in 1.0 M KOH using carbon rod as the counter electrode at room temperature.

### The ECSAs

ECSAs were calculated by dividing *C*_dl_ by a specific capacitance (*C*_s_, assumed as 0.04 mF cm^−2^ in 1.0 M KOH).

### Material characterization

Powder XRD patterns were recorded using a Rigaku D/Max-2200 PC diffractometer in the diffraction angle range 2θ = 5° to 80° with Cu Ka radiation (λ = 1.5418 Å) at 40 kV, 40 mA. XPS was measured on a PerkinElmer model PHI 5500 XPS system with a resolution of 0.3 to 0.5 eV from a monochromated aluminum anode x-ray source with Mo Kα radiation (1486.6 eV). All XPS spectra were calibrated using the C 1s peak of carbon present at 284.80 eV. Field-emission SEM was characterized by a Nona-Nano SEM450 at the accelerating voltage of 5 kV. TEM, HRTEM, and high-angle annular dark-field scanning transmission electron microscopy–energy dispersive x-ray spectroscopy (HAADF-STEM-EDX) were performed on Tecnai G2 TF30 operating at 300 kV. Fourier transform infrared (FTIR) spectra (KBr pellets) were conducted on a Thermo Electron NEXUS 670 FTIR spectrometer.

### In situ Raman measurements

The electrochemical Raman measurements were carried out on a confocal LabRAM Soleil Raman Microscope Raman system (HORIBA FRANCE SAS). An He-Ne laser with 532-nm excitation wavelength and a 50× microscope objective with a numerical aperture of 0.55 were used in all measurements. Raman frequency was calibrated by a standard silicon (Si) wafer to 520.7 cm^−1^ during each experiment. In situ electrochemical Raman experiments were used in a Raman cell, and an electrochemical workstation CHI 760E potentiostat was used to control the potential. Raman curves were recorded ranging from 50 to 2000 cm^−1^. Operando Raman spectra were performed using a homemade open spectro-electrochemical cell consisting of a three-electrode system. A calibrated Hg/HgO (1 M KOH) electrode and a Pt plate served as the reference and counter electrodes, respectively.

### Methods

#### DFT calculations

DFT calculations with Hubbard-*U* corrections were performed using the Vienna Ab Initio Simulation Package (version 5.4.4) (*46*). The electronic exchange and correlation interactions were described by the Perdew-Burke-Ernzerhof (PBE) form of generalized gradient approximation (*46*) and the projector-augmented wave method (*47*) were used. We use the effective Hubbard-*U* (*U*_eff_ = *U* − *J*) parameters from Wei *et al.* (*42*), Baek *et al.* (*48*) and Winther *et al.* (*49*) that are optimized to match the M(OH)_2_/MOOH redox features, i.e., 4.38 (Mo), 3.0 (Zn), 4.3 (Fe), 3.32 (Co), and 5.5 (Ni). The β-MOOH (*R*$\bar{3}$*m* space group) with (01$\bar{1}$2)-terminated surface was selected as our slab model, and a 3 × 2 × 3 supercell was established. The bottom two layers are kept as NiOOH, and the top layer is doped with Mo, Zn, Fe, and Co. A vacuum layer of 15 Å was introduced in the *z* direction to prevent interactions between periodic images. During optimization, the two bottom layers were fixed to mimic bulk structures, while the top layer (and the adsorbates) was fully relaxed. The electronic wave function was expanded in plane wave basis sets with a cutoff energy of 450 eV with a Monkhorst-Pack (*50*) 2 × 2 × 1 *k*-point grid. The energy and force convergence criteria were set to be 10^−5^ eV and 0.05 eV Å^−1^, respectively. The dispersion corrected PBE-D3 method in the Grimme’s scheme (*51*) was used to include the van der Waals interactions.

Because of the strong *d-d* Coulomb interaction, the *d* orbitals are split into a filled LHB and an empty UHB (*1*). The centers of LHB and UHB can be calculated from the PDOS distribution below and above Efermi, respectively

$${\bar{\varepsilon }}_{\text{LHB}}=\frac{\int_{-\infty }^{0}\varepsilon \text{DOS}\left(\varepsilon \right)d\varepsilon }{\int_{-\infty }^{0}\text{DOS}\left(\varepsilon \right)d\varepsilon }$$ (1)

$${\bar{\varepsilon }}_{\text{UHB}}=\frac{\int_{0}^{+\infty }\varepsilon \text{DOS}\left(\varepsilon \right)d\varepsilon }{\int_{0}^{+\infty }\text{DOS}\left(\varepsilon \right)d\varepsilon }$$ (2)

where ε represents the energy level and DOS(ε) denotes the density of states at this energy level. Because of the 3*d* states of Zn being completely filled, Zn does not have an UHB. Bader charge analysis is performed using the algorithm developed by Henkelman *et al.* (*52*).

The computational hydrogen electrode model was applied to simulate the OER pathway and determine the Gibbs free energy diagrams for different sites (*53*). Both AEM and LOM pathways are considered to identify the active sites. For the AEM pathway, the following four proton-electron transfer steps are considered

$${}^{*}+{\text{OH}}^{-}\to {}^{*}\text{OH}+e^{-}$$ (3)

$${}^{*}\text{OH}+{\text{OH}}^{-}\to {}^{*}O+H_{2}O+e^{-}$$ (4)

$${}^{*}O+{\text{OH}}^{-}\to {}^{*}\text{OOH}+e^{-}$$ (5)

$${}^{*}\text{OOH}+{\text{OH}}^{-}\to {}^{*}+O_{2}+H_{2}O+e^{-}$$ (6)

where * denotes the active site of the catalyst surface and *O, *OH, and *OOH indicate the intermediate species adsorbed on the active site. For each of the four steps, the Gibbs free energy change (Δ*G*) of the intermediate was defined as

ΔG=ΔE+ΔZPE–TΔS (7)

where Δ*E* is the electronic energy difference; ΔZPE and Δ*S* are the difference of zero-point energies and the change of entropy, respectively, which can be obtained by vibrational frequency analysis; and *T* is 298.15 K.

The following formula was used to calculate overpotential (η) to compare OER activity

$$\eta =\text{max}\left[\Delta G_{1},\Delta G_{2},\Delta G_{3},\Delta G_{4}\right]/e–1.23\;V$$ (8)

For the LOM pathway, the single-site LOM mechanism is considered, which involve the following four steps

$${}^{*}+{\text{OH}}^{-}\to {}^{*}\text{OH}+e^{-}$$ (9)

$${}^{*}\text{OH}+{\text{OH}}^{-}\to {}^{*}O_{\text{vac}}+H_{2}O+O_{2}\left(g\right)+e^{-}$$ (10)

$${}^{*}O_{\text{vac}}+{\text{OH}}^{-}\to {}^{*}H+e^{-}$$ (11)

$${}^{*}H+{\text{OH}}^{-}\to {}^{*}+H_{2}O+e^{-}$$ (12)

In this scenario, a hydroxyl group initially attacks the oxide surface, bonding with a lattice oxygen atom. The surface hydroxyl then loses a proton, resulting in the desorption of O_2_ and the creation of a lattice oxygen vacancy (*O_vac_). Subsequently, another hydroxyl group fills the vacancy, and following a second deprotonation step, the surface is completely restored. Following Eqs. 7 and 8, the LOM overpotential and Gibbs free energy diagrams can be derived from the four OER steps.

## Supplementary Materials

### This PDF file includes:

Supplementary Text Figs. S1 to S18 Tables S1 to S9 References

## References

[1] Z. F. Huang, J. J. Song, Y. H. Du, S. B. Xi, S. Dou, J. M. V. Nsanzimana, C. Wang, Z. C. J. Xu, X. Wang, Chemical and structural origin of lattice oxygen oxidation in Co-Zn oxyhydroxide oxygen evolution electrocatalysts. Nat. Energy 4, 329–338 (2019).

[2] H. B. Tao, Y. H. Xu, X. Huang, J. Z. Chen, L. J. Pei, J. M. Zhang, J. G. G. Chen, B. Liu, A general method to probe oxygen evolution intermediates at operating conditions. Joule 3, 1498–1509 (2019).

[3] R. He, L. L. Yang, Y. Zhang, D. Jiang, S. Lee, S. Horta, Z. F. Liang, X. Lu, A. O. Moghaddam, J. S. Li, M. Ibáñez, Y. Xu, Y. T. Zhou, A. Cabot, A 3d-4d-5d high entropy alloy as a bifunctional oxygen catalyst for robust aqueous zinc-air batteries. Adv. Mater. 35, 202303719 (2023). 10.1002/adma.202303719 37487245

[4] Q. W. Zhang, Y. X. Hu, H. F. Wu, X. R. Zhao, M. L. Wang, S. H. Wang, R. H. Feng, Q. Chen, F. Song, M. W. Chen, P. Liu, Entropy-stabilized multicomponent porous spinel nanowires of NiFe_x_O_4_ (x = Fe, Ni, Al, Mo, Co, Cr) for efficient and durable electrocatalytic oxygen evolution reaction in alkaline medium. ACS Nano 17, 1485–1494 (2023). 10.1021/acsnano.2c10247 36630198

[5] X. Wang, H. Zhong, S. Xi, W. S. V. Lee, J. Xue, Understanding of oxygen redox in the oxygen evolution reaction. Adv. Mater. 34, 202107956 (2022). 10.1002/adma.202107956 35853837

[6] Y. Mei, Y. Feng, C. Zhang, Y. Zhang, Q. Qi, J. Hu, High-entropy alloy with Mo-coordination as efficient electrocatalyst for oxygen evolution reaction. ACS Catal. 12, 10808–10817 (2022).

[7] M. Lu, Y. Zheng, Y. Hu, B. L. Huang, D. G. Ji, M. Z. Sun, J. Y. Li, Y. Peng, R. Si, P. X. Xi, C. H. Yan, Artificially steering electrocatalytic oxygen evolution reaction mechanism by regulating oxygen defect contents in perovskites. Sci. Adv. 8, eabq3563 (2022). 35905191 10.1126/sciadv.abq3563 PMC9337758

[8] Z. Y. He, J. Zhang, Z. H. Gong, H. Lei, D. Zhou, N. A. Zhang, W. J. Mai, S. J. Zhao, Y. Chen, Activating lattice oxygen in NiFe-based (Oxy)hydroxide for water electrolysis. Nat. Commun. 13, 2191 (2022). 35449165 10.1038/s41467-022-29875-4 PMC9023528

[9] A. K. Tomar, U. N. Pan, N. H. Kim, J. H. Lee, Enabling lattice oxygen participation in a triple perovskite oxide electrocatalyst for the oxygen evolution reaction. ACS Energy Lett. 8, 565–573 (2022).

[10] K. Huang, J. Y. Xia, Y. Lu, B. W. Zhang, W. C. Shi, X. Cao, X. Y. Zhang, L. M. Woods, C. C. Han, C. J. Chen, T. Wang, J. S. Wu, Y. Z. Huang, Self-reconstructed spinel surface structure enabling the long-term stable hydrogen evolution reaction/oxygen evolution reaction efficiency of feconiru high-entropy alloyed electrocatalyst. Adv. Sci. 10, 202300094 (2023). 10.1002/advs.202300094 PMC10190517 36950752

[11] Y. G. Yao, Z. N. Huang, P. F. Xie, S. D. Lacey, R. J. Jacob, H. Xie, F. J. Chen, A. M. Nie, T. C. Pu, M. Rehwoldt, D. W. Yu, M. R. Zachariah, C. Wang, R. Shahbazian-Yassar, J. Li, L. B. Hu, Carbothermal shock synthesis of high-entropy-alloy nanoparticles. Science 359, 1489–1494 (2018). 29599236 10.1126/science.aan5412

[12] Z. Jing, Y. Guo, Q. Wang, X. Yan, G. Yue, Z. Li, H. Liu, R. Qin, C. Zhong, M. Li, D. Xu, Y. Yao, Y. Yao, M. Shuai, Ambient hydrogenation of solid aromatics enabled by a high entropy alloy nanocatalyst. Nat. Commun. 15, 5806 (2024). 38987569 10.1038/s41467-024-50009-5 PMC11236972

[13] L. L. Yu, K. Z. Zeng, C. H. Li, X. R. Lin, H. W. Liu, W. H. Shi, H. J. Qiu, Y. F. Yuan, Y. G. Yao, High-entropy alloy catalysts: From bulk to nano toward highly efficient carbon and nitrogen catalysis. Carbon Energy 4, 731–761 (2022).

[14] L. He, M. Li, L. Qiu, S. Geng, Y. Liu, F. Tian, M. Luo, H. Liu, Y. Yu, W. Yang, S. Guo, Single-atom Mo-tailored high-entropy-alloy ultrathin nanosheets with intrinsic tensile strain enhance electrocatalysis. Nat. Commun. 15, 2290–2290 (2024). 38480686 10.1038/s41467-024-45874-z PMC10937678

[15] Z. J. Li, S. Q. Ji, C. Wang, H. X. Liu, L. P. Leng, L. Du, J. C. Gao, M. Qiao, J. H. Horton, Y. Wang, Geometric and electronic engineering of atomically dispersed copper-cobalt diatomic sites for synergistic promotion of bifunctional oxygen electrocatalysis in zinc-air batteries. Adv. Mater. 35, 202300905 (2023). 10.1002/adma.202300905 37040668

[16] M. Miao, J. Pan, T. He, Y. Yan, B. Y. Xia, X. Wang, Molybdenum carbide-based electrocatalysts for hydrogen evolution reaction. Chem. Eur. J. 23, 10947–10961 (2017). 28474426 10.1002/chem.201701064

[17] Y. G. Yao, Z. N. Huang, L. A. Hughes, J. L. Gao, T. Y. Li, D. Morris, S. E. Zeltmann, B. H. Savitzky, C. Ophus, Y. Z. Finfrock, Q. Dong, M. L. Jiao, Y. M. Mao, M. F. Chi, P. Zhang, J. Li, A. M. Minor, R. Shahbazian-Yassar, L. B. Hu, Extreme mixing in nanoscale transition metal alloys. Matter 4, 2340–2353 (2021).

[18] W. H. Shi, Z. Z. Li, Z. H. Gong, Z. H. Liang, H. W. Liu, Y. C. Han, H. T. Niu, B. Song, X. D. Chi, J. H. Zhou, H. Wang, B. Y. Xia, Y. G. Yao, Z. Q. Tian, Transient and general synthesis of high-density and ultrasmall nanoparticles on two-dimensional porous carbon via coordinated carbothermal shock. Nat. Commun. 14, 2294 (2023). 37085505 10.1038/s41467-023-38023-5 PMC10121605

[19] Y. X. Wang, Y. Zhang, P. Y. Xing, X. Q. Li, Q. Y. Du, X. Q. Fan, Z. B. Cai, R. Yin, Y. G. Yao, W. T. Gan, Self-encapsulation of high-entropy alloy nanoparticles inside carbonized wood for highly durable electrocatalysis. Adv. Mater. 36, 02391 (2024). 10.1002/adma.202402391 38669588

[20] B. Talluri, K. Yoo, J. Kim, High entropy spinel metal oxide (CoCrFeMnNi)_3_O_4_ nanoparticles as novel efficient electrocatalyst for methanol oxidation and oxygen evolution reactions. J. Environ. Chem. Eng. 10, 106932 (2022).

[21] L. Sharma, N. K. Katiyar, A. Parui, R. Das, R. Kumar, C. S. Tiwary, A. K. Singh, A. Halder, K. Biswas, Low-cost high entropy alloy (HEA) for high-efficiency oxygen evolution reaction (OER). Nano Res. 15, 4799–4806 (2022).

[22] P. Li, X. Wan, J. Su, W. Liu, Y. Guo, H. Yin, D. Wang, A single-phase FeCoNiMnMo high-entropy alloy oxygen evolution anode working in alkaline solution for over 1000 h. ACS Catal. 12, 11667–11674 (2022).

[23] L. He, N. Wang, B. Sun, L. Zhong, M. Yao, W. Hu, S. Komarneni, High-entropy FeCoNiMn (Oxy)hydroxide as high-performance electrocatalyst for OER and boosting clean carrier production under quasi-industrial condition. J. Clean. Prod. 356, 131680 (2022).

[24] Z. Jin, J. Lyu, Y.-L. Zhao, H. Li, X. Lin, G. Xie, X. Liu, J.-J. Kai, H.-J. Qiu, Rugged high-entropy alloy nanowires with in situ formed surface spinel oxide as highly stable electrocatalyst in zn–air batteries. ACS Mater. Lett. 2, 1698–1706 (2020).

[25] W. Dai, T. Lu, Y. Pan, Novel and promising electrocatalyst for oxygen evolution reaction based on MnFeCoNi high entropy alloy. J. Power Sources 430, 104–111 (2019).

[26] X. H. Zhao, Z. M. Xue, W. J. Chen, Y. Q. Wang, T. C. Mu, Eutectic synthesis of high-entropy metal phosphides for electrocatalytic water splitting. Chemsuschem 13, 2038–2042 (2020). 31981404 10.1002/cssc.202000173

[27] J. Tang, J. L. Xu, Z. G. Ye, X. B. Li, J. M. Luo, Microwave sintered porous CoCrFeNiMo high entropy alloy as an efficient electrocatalyst for alkaline oxygen evolution reaction. J. Mater. Sci. Technol. 79, 171–177 (2021).

[28] Z. Ding, J. Bian, S. Shuang, X. Liu, Y. Hu, C. Sun, Y. Yang, High entropy intermetallic-oxide core-shell nanostructure as superb oxygen evolution reaction catalyst. Adv. Sustain. Syst. 4, 1900105 (2020).

[29] T. Zhang, J. Li, B. Zhang, G. Wang, K. Jiang, Z. Zheng, J. Shen, High-entropy alloy CuCrFeNiCoP film of Cu-based as high-efficiency electrocatalyst for water splitting. J. Alloys Compd. 969, 172439 (2023).

[30] F. M. Liu, M. Yu, X. Chen, J. H. Li, H. H. Liu, F. Y. Cheng, Defective high-entropy rocksalt oxide with enhanced metal-oxygen covalency for electrocatalytic oxygen evolution. Chinese J. Catal. 43, 122–129 (2022).

[31] X. H. Zhao, Z. M. Xue, W. J. Chen, X. Y. Bai, R. F. Shi, T. C. Mu, Ambient fast, large-scale synthesis of entropy-stabilized metal-organic framework nanosheets for electrocatalytic oxygen evolution. J. Mater. Chem. A 7, 26238–26242 (2019).

[32] X. D. Cui, B. L. Zhang, C. Y. Zeng, S. M. Guo, Electrocatalytic activity of high-entropy alloys toward oxygen evolution reaction. MRS Commun. 8, 1230–1235 (2018).

[33] H. J. Qiu, G. Fang, J. J. Gao, Y. R. Wen, J. Lv, H. L. Li, G. Q. Xie, X. J. Liu, S. H. Sun, Noble metal-free nanoporous high-entropy alloys as highly efficient electrocatalysts for oxygen evolution reaction. ACS Mater. Lett. 1, 526–533 (2019).

[34] L. H. Liu, N. Li, M. Han, J. R. Han, H. Y. Liang, Scalable synthesis of nanoporous high entropy alloys for electrocatalytic oxygen evolution. Rare Met. 41, 125–131 (2022).

[35] R. He, L. L. Yang, Y. Zhang, X. Wang, S. Lee, T. Zhang, L. X. Li, Z. F. Liang, J. W. Chen, J. S. Li, A. O. Moghaddam, J. Llorca, M. Ib, J. Arbiol, Y. Xu, A. Cabot, A CrMnFeCoNi high entropy alloy boosting oxygen evolution/reduction reactions and zinc-air battery performance. Energy Storage Mater. 58, 287–298 (2023).

[36] L. Y. Yi, S. M. Xiao, Y. P. Wei, D. Z. Li, R. F. Wang, S. F. Guo, W. H. Hu, Free-standing high-entropy alloy plate for efficient water oxidation catalysis: Structure/composition evolution and implication of high-valence metals. Chem. Eng. J. 469, 144015 (2023).

[37] R. R. Zhang, L. Pan, B. B. Guo, Z. F. Huang, Z. X. Chen, L. Wang, X. W. Zhang, Z. Y. Guo, W. Xu, K. P. Loh, J. J. Zou, Tracking the role of defect types in Co_3_O_4_ structural evolution and active motifs during oxygen evolution reaction. J. Am. Chem. Soc. 145, 2271–2281 (2023). 36654479 10.1021/jacs.2c10515

[38] C. J. Hu, Y. F. Hu, C. H. Fan, L. Yang, Y. T. Zhang, H. X. Li, W. Xie, Surface-enhanced raman spectroscopic evidence of key intermediate species and role of NiFe dual-catalytic center in water oxidation. Angew. Chem. Int. Ed. 60, 19774–19778 (2021). 10.1002/anie.202103888 34184371

[39] C. Wang, P. L. Zhai, M. Y. Xia, W. Liu, J. F. Gao, L. C. Sun, J. A. Hou, Identification of the origin for reconstructed active sites on oxyhydroxide for oxygen evolution reaction. Adv. Mater. 35, 09307 (2023). 10.1002/adma.202209307 36408935

[40] H. Q. Chu, R. J. Li, P. P. Feng, D. Y. Wang, C. X. Li, Y. L. Yu, M. Yang, Ligands defect-induced structural self-reconstruction of Fe-Ni-Co-hydroxyl oxides with crystalline/amorphous heterophase from a 2D metal-organic framework for an efficient oxygen evolution reaction. ACS Catal. 14, 1553–1566 (2024).

[41] E. Fabbri, M. Nachtegaal, T. Binninger, X. Cheng, B. J. Kim, J. Durst, F. Bozza, T. Graule, R. Schäublin, L. Wiles, M. Pertoso, N. Danilovic, K. E. Ayers, T. J. Schmidt, Dynamic surface self-reconstruction is the key of highly active perovskite nano-electrocatalysts for water splitting. Nat. Mater. 16, 925–931 (2017). 28714982 10.1038/nmat4938

[42] L. Z. Wei, M. D. Hossain, M. J. Boyd, J. Aviles-Acosta, M. E. Kreider, A. C. Nielander, M. B. Stevens, T. F. Jaramillo, M. Bajdich, C. Hahn, Insights into active sites and mechanisms of benzyl alcohol oxidation on nickel-iron oxyhydroxide electrodes. ACS Catal. 13, 4272–4282 (2023).

[43] Y. M. Sun, H. B. Liao, J. R. Wang, B. Chen, S. N. Sun, S. J. H. Ong, S. B. Xi, C. Z. Diao, Y. H. Du, J. O. Wang, M. B. H. Breese, S. Z. Li, H. Zhang, Z. C. J. Xu, Covalency competition dominates the water oxidation structure-activity relationship on spinel oxides. Nat. Catal. 3, 959–959 (2020).

[44] B. Zhang, L. Wang, Z. Cao, S. M. Kozlov, F. P. G. de Arquer, C. T. Dinh, J. Li, Z. Y. Wang, X. L. Zheng, L. S. Zhang, Y. Z. Wen, O. Voznyy, R. Comin, P. De Luna, T. Regier, W. L. Bi, E. E. Alp, C. W. Pao, L. R. Zheng, Y. F. Hu, Y. J. Ji, Y. Y. Li, Y. Zhang, L. Cavallo, H. S. Peng, E. H. Sargent, High-valence metals improve oxygen evolution reaction performance by modulating 3D metal oxidation cycle energetics. Nat. Catal. 3, 985–992 (2020).

[45] J. Wang, J. Zhang, L. Zhang, L. Chen, G. He, H. Jiang, Modulating metal-oxygen interactions of high-entropy oxide electrocatalysts enables highly-active and ultra-stable water oxidation. Appl. Catal. B. Environ. 342, 123382 (2024).

[46] J. P. Perdew, K. Burke, M. Ernzerhof, Generalized gradient approximation made simple. Phys. Rev. Lett. 77, 3865–3868 (1996). 10062328 10.1103/PhysRevLett.77.3865

[47] P. E. Blöchl, Projector augmented-wave method. Phys. Rev. B 50, 17953–17979 (1994). 10.1103/physrevb.50.17953 9976227

[48] J. Baek, M. D. Hossain, P. Mukherjee, J. H. Lee, K. T. Winther, J. Leem, Y. Jiang, W. C. Chueh, M. Bajdich, X. L. Zheng, Synergistic effects of mixing and strain in high entropy spinel oxides for oxygen evolution reaction. Nat. Commun. 14, 5936 (2023). 37741823 10.1038/s41467-023-41359-7 PMC10517924

[49] K. Winther, M. Hoffmann, O. Mamun, J. Boes, M. Bajdich, T. Bligaard, Catalysis-hub.Org: An open electronic structure database for surface reactions and catalytic materials. Abstr. Pap. Am. Chem. Soc. 257, 88 (2019). 10.1038/s41597-019-0081-y PMC6538711 31138816

[50] H. J. Monkhorst, J. D. Pack, Special points for brillouin-zone integrations. Phys. Rev. B 13, 5188–5192 (1976).

[51] S. Grimme, J. Antony, S. Ehrlich, H. Krieg, A consistent and accurate ab initio parametrization of density functional dispersion correction (DFT-D) for the 94 elements h-pu. J. Chem. Phys. 132, 154104 (2010). 20423165 10.1063/1.3382344

[52] G. Henkelman, A. Arnaldsson, H. Jónsson, A fast and robust algorithm for bader decomposition of charge density. Comput. Mater. Sci. 36, 354–360 (2006).

[53] J. K. Nørskov, J. Rossmeisl, A. Logadottir, L. Lindqvist, J. R. Kitchin, T. Bligaard, H. Jónsson, Origin of the overpotential for oxygen reduction at a fuel-cell cathode. J. Phys. Chem. B. 108, 17886–17892 (2004). 10.1021/jp047349j 39682080

[54] J. Hu, L. Cao, Z. Wang, J. Liu, J. Zhang, Y. Cao, Z. Lu, H. Cheng, Hollow high-entropy metal organic framework derived nanocomposite as efficient electrocatalyst for oxygen reduction reaction. Compos. Commun. 27, 100866 (2021).

[55] Z.-J. Zhang, J.-P. Guo, S.-H. Sun, Q. Sun, Y.-W. Zhao, Y.-F. Zhang, Z.-Y. Yu, C.-S. Li, Y. Sun, M.-M. Zhang, Y. Jiang, Optimized valence state of Co and Ni in high-entropy alloy for high active-stable oer. Rare Met. 42, 3607–3613 (2023).

[56] P. Yang, Y. An, C. Feng, Y. Liu, S. Liu, L. Gao, Y. Zhou, X. Li, P. Li, F. Zeng, Heterogeneous high-entropy catalyst nanoparticles for oxygen evolution reaction: Impact of oxygen and fluorine introduction. Int. J. Hydrogen Energy 51, 1218–1228 (2024).

[57] G. Raj, R. Nandan, K. Kumar, D. B. Gorle, A. B. Mallya, S. M. Osman, J. Na, Y. Yamauchi, K. K. Nanda, High entropy alloying strategy for accomplishing quintuple-nanoparticles grafted carbon towards exceptional high-performance overall seawater splitting. Mater. Horiz. 10, 5032–5044 (2023). 37649459 10.1039/d3mh00453h

[58] J. X. Yang, B. H. Dai, C. Y. Chiang, I. C. Chiu, C. W. Pao, S. Y. Lu, I. Y. Tsao, S. T. Lin, C. T. Chiu, J. W. Yeh, P. C. Chang, W. H. Hung, Rapid fabrication of high-entropy ceramic nanomaterials for catalytic reactions. ACS Nano 15, 12324–12333 (2021). 34269062 10.1021/acsnano.1c04259

[59] H. Y. Wang, R. Wei, X. M. Li, X. L. Ma, X. G. Hao, G. Q. Guan, Nanostructured amorphous Fe_29_Co_27_Ni_23_Si_9_B_12_ high-entropy-alloy: An efficient electrocatalyst for oxygen evolution reaction. J. Mater. Sci. Technol. 68, 191–198 (2021).

[60] S. Q. Zhao, H. Y. Wu, R. Yin, X. N. Wang, H. Z. Zhong, Q. Fu, W. J. Wan, T. Cheng, Y. Shi, G. X. Cai, C. Z. Jiang, F. Ren, Preparation and electrocatalytic properties of (FeCrCoNiAl_0.1_)O_x_ high-entropy oxide and NiCo-(FeCrCoNiAl_0.1_)O_x_ heterojunction films. J. Alloys Compd. 868, 159108 (2021).

[61] P. Y. Ma, S. C. Zhang, M. T. Zhang, J. F. Gu, L. Zhang, Y. C. Sun, W. Ji, Z. Y. Fu, Hydroxylated high-entropy alloy as highly efficient catalyst for electrochemical oxygen evolution reaction. Sci. China Mater. 63, 2613–2619 (2020).

[62] K. Z. Gu, X. Y. Zhu, D. D. Wang, N. N. Zhang, G. Huang, W. Li, P. Long, J. Tian, Y. Q. Zou, Y. Y. Wang, R. Chen, S. Y. Wang, Ultrathin defective high-entropy layered double hydroxides for electrochemical water oxidation. J. Energy Chem. 60, 121–126 (2021).

[63] K. Huang, B. Zhang, J. Wu, T. Zhang, D. Peng, X. Cao, Z. Zhang, Z. Li, Y. Huang, Exploring the impact of atomic lattice deformation on oxygen evolution reactions based on a sub-5 nm pure face-centred cubic high-entropy alloy electrocatalyst. J. Mater. Chem. A 8, 11938–11947 (2020).

[64] Z. Chen, J. Wen, C. Wang, X. Kang, Convex cube-shaped Pt_34_Fe_5_Ni_2_Cu_31_Mo_9_Ru high entropy alloy catalysts toward high-performance multifunctional electrocatalysis. Small 18, 2204255 (2022). 10.1002/smll.202204255 36161488

[65] Y. M. Zhang, J. L. Kang, H. A. Xie, H. X. Yin, Z. J. Zhang, E. Z. Liu, L. Y. Ma, B. Chen, J. W. Sha, L. H. Qian, W. B. Hu, C. N. He, N. Q. Zhao, Boosting the oxygen evolution of high-entropy (Oxy)hydroxide epitaxially grown on high entropy alloy by lattice oxygen activation. Appl. Catal. B. Environ. 341, 123331 (2024).

[66] J. H. Cha, S. H. Cho, D. H. Kim, D. Jeon, S. Park, J. W. Jung, I. Kim, S. Y. Choi, Flash-thermal shock synthesis of high-entropy alloys toward high-performance water splitting. Adv. Mater. 35, 202305222 (2023). 10.1002/adma.202305222 37607534

[67] A. N. Nguyen, N. M. Tran, H. Yoo, Direct growth and post-treatment of zeolitic imidazolate framework-67 on carbon paper: An effective and stable electrode system for electrocatalytic reactions. J. Mater. Chem. A 10, 20770–20778 (2022).

[68] Y. Li, Q. Zhang, X. Zhao, H. Wu, X. Wang, Y. Zeng, Q. Chen, M. Chen, P. Liu, Vapor phase dealloying derived nanoporous Co@CoO/RuO_2_ composites for efficient and durable oxygen evolution reaction. Adv. Funct. Mater. 33, 202214124 (2023).

[69] W. Zhang, M. Niu, J. Yu, S. Li, Y. Wang, K. Zhou, Mechanochemical post-synthesis of metal-organic framework-based pre-electrocatalysts with surface Fe-O-Ni/Co bonding for highly efficient oxygen evolution. Adv. Funct. Mater. 33, 202302014 (2023).

[70] Y. F. Cui, S. D. Jiang, Q. Fu, R. Wang, P. Xu, Y. Sui, X. J. Wang, Z. L. Ning, J. F. Sun, X. Sun, A. Nikiforov, B. Song, Cost-effective high entropy core–shell fiber for stable oxygen evolution reaction at 2 A cm^−2^. Adv. Funct. Mater. 33, 202306889 (2023).

[71] K. Yu, H. Yang, H. Zhang, H. Huang, Z. Wang, Z. Kang, Y. Liu, P. W. Menezes, Z. Chen, Immobilization of oxyanions on the reconstructed heterostructure evolved from a bimetallic oxysulfide for the promotion of oxygen evolution reaction. Nanomicro. Lett. 15, 186 (2023). 37515724 10.1007/s40820-023-01164-9 PMC10387036

[72] M. Li, X. Wang, K. Liu, Z. Zhu, H. Guo, M. Li, H. Du, D. Sun, H. Li, K. Huang, Y. Tang, G. Fu, Ce-induced differentiated regulation of Co sites via gradient orbital coupling for bifunctional water-splitting reactions. Adv. Energy Mater. 13, 202301162 (2023).

[73] S. Wang, W. Huo, H. Feng, Z. Xie, J. K. Shang, E. V. Formo, P. H. C. Camargo, F. Fang, J. Jiang, Enhancing oxygen evolution reaction performance in prussian blue analogues: Triple-play of metal exsolution, hollow interiors, and anionic regulation. Adv. Mater. 35, e2304494 (2023). 37473821 10.1002/adma.202304494

