# Supplementary material for: MoZn-based high entropy alloy catalysts enabled dual activation and stabilization in alkaline oxygen evolution
Source: Sci Adv. 2024 Nov 20;10(47):eadq6758. doi: 10.1126/sciadv.adq6758 (PMC11639200; doi:10.1126/sciadv.adq6758)
Supplement: Supplementary file 1 — Supplementary Text Figs. S1 to S18 Tables S1 to S9 References [file sciadv.adq6758_sm.pdf]

Science Advances
AAAS

Supplementary Materials for

MoZn-based high entropy alloy catalysts enabled dual activation and stabilization in alkaline oxygen evolution

Yunjie Mei *et al.*

Corresponding author: Yonggang Yao, yaoyg@hust.edu.cn; Bao Yu Xia, byxia@hust.edu.cn; Yuhua Wang, wangyuhua@wust.edu.cn

Sci. Adv. **10**, eadq6758 (2024)
DOI: 10.1126/sciadv.adq6758

This PDF file includes:

Supplementary Text
Figs. S1 to S18
Tables S1 to S9
References

Supporting Figures

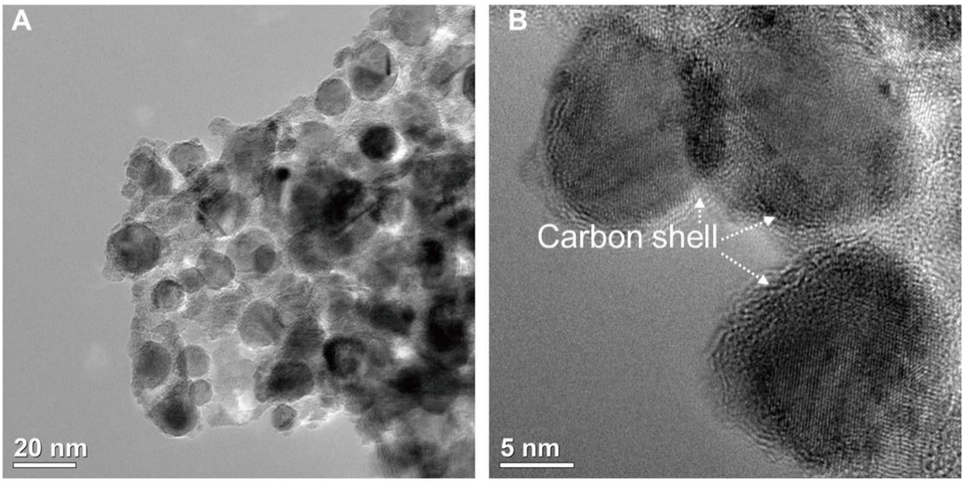

Fig. S1. TEM characterizations. (A-B) The HRTEM images of ST-HEA.

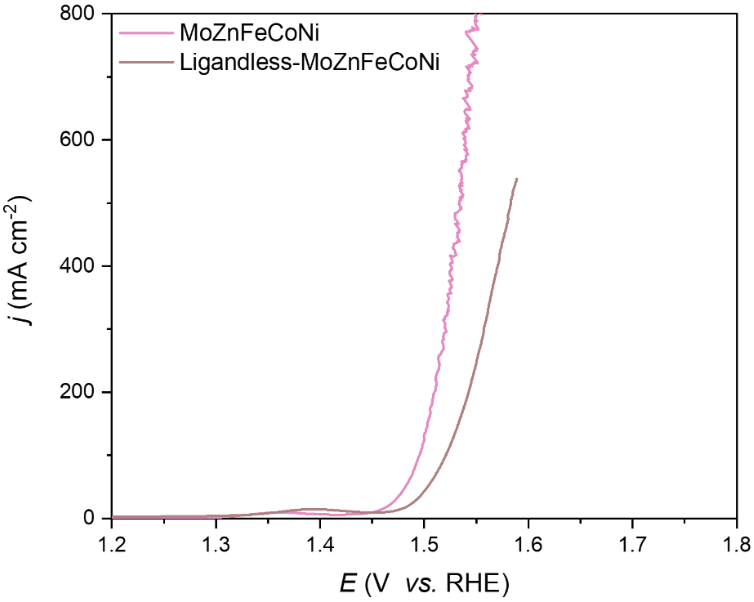

Fig. S2. **Polarization curves.** Comparison of electrochemical performances of MoZnFeCoNi by adding/no ligands during shock synthesis (i.e., with and without carbon shell).

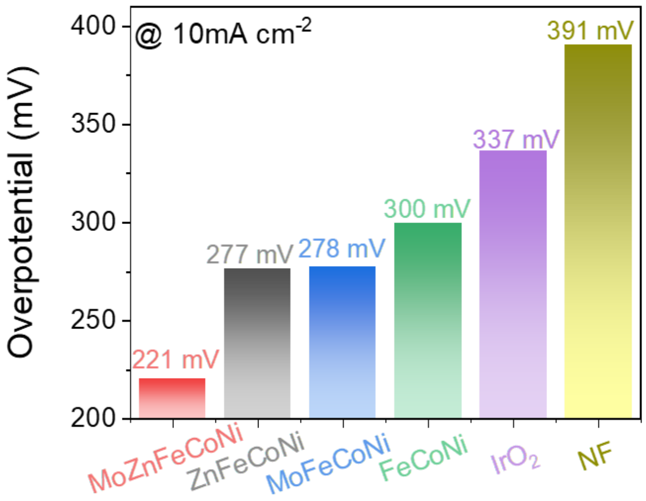

Fig. S3. Comparison of overpotential. The comparison of the overpotential at 10 mA cm^{-2} of the MoZnFeCoNi, ZnFeCoNi, MoFeCoNi and FeCoNi catalysts in 1.0 M KOH solution at room temperature.

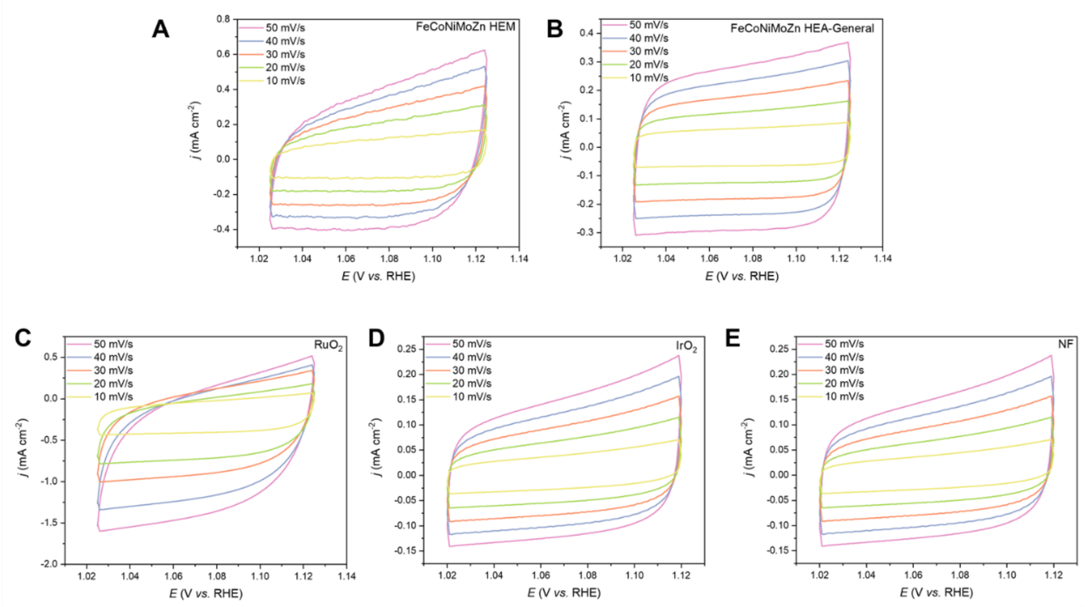

Fig. S4. Cyclic voltammograms (CV) of catalysts of different synthetic modes. (A) FeCoNiMoZn-HEM, (B) FeCoNiMoZn-General, (C) RuO_2, (D) IrO_2, (E) NF.

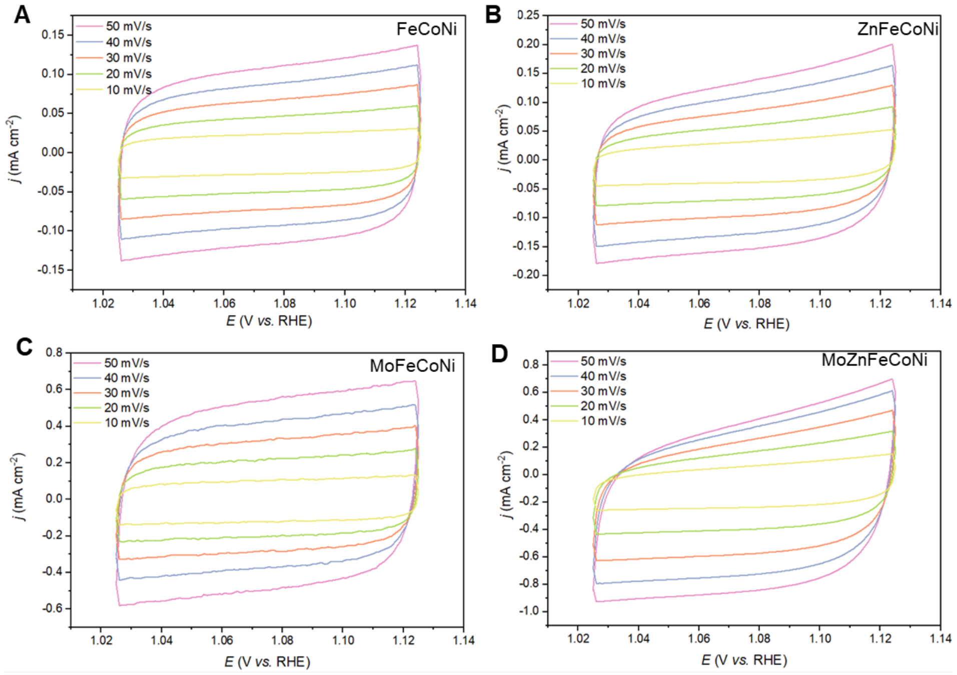

Fig. S5. Cyclic voltammograms curve. (A) FeCoNi and (B) ZnFeCoNi, (C) MoFeCoNi and (D) MoZnFeCoNi (NF) in 1.0 M KOH.

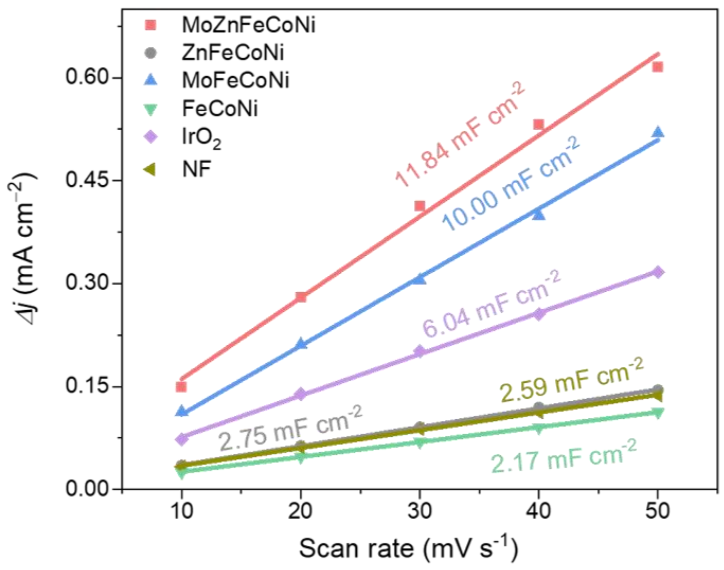

Fig. S6. Electrochemical double-layer capacitance analyses. Charging current density differences plotted against scan rate of MoZnFeCoNi, ZnFeCoNi, MoFeCoNi, FeCoNi, IrO_2 and NF.

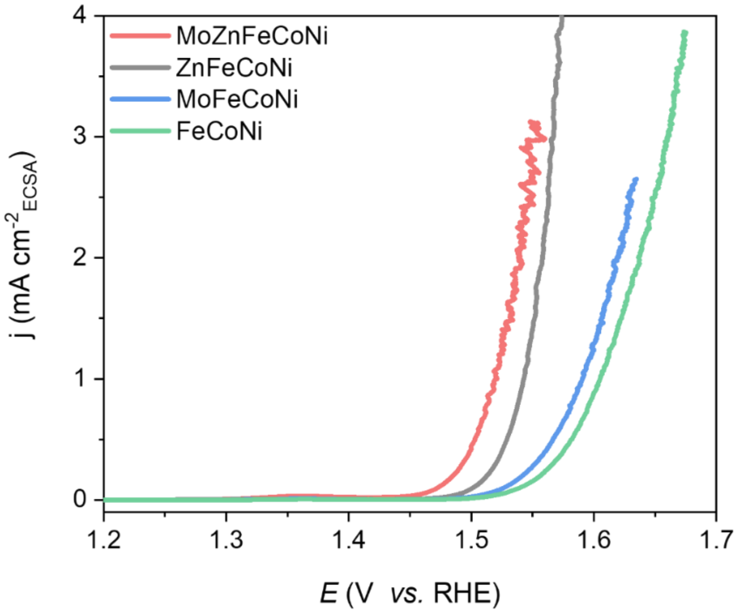

Fig. S7. Polarization curves. Polarization curves (current normalized) of the MoZnFeCoNi, ZnFeCoNi, MoFeCoNi, and FeCoNi.

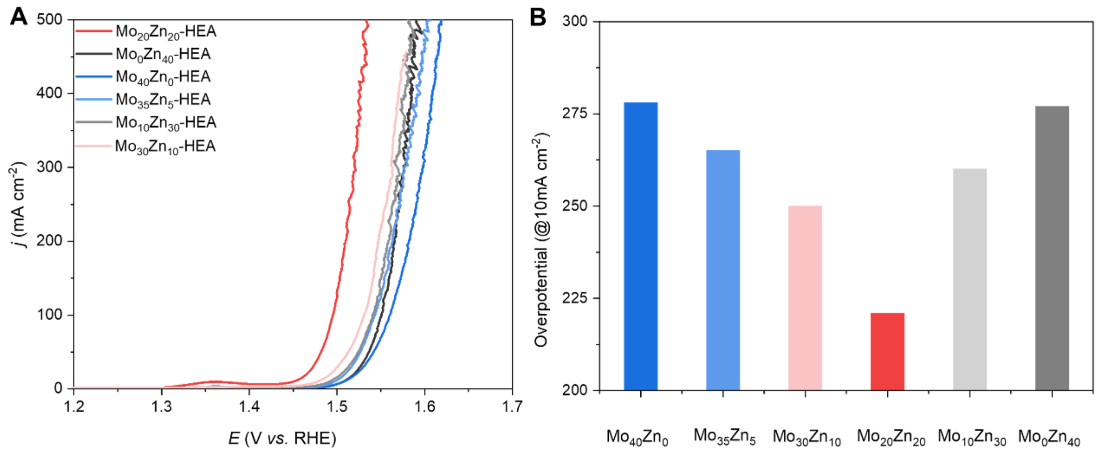

Fig. S8. Polarization curves and comparison of overpotential. The electrocatalysts with different Zn content of (A) electrochemical polarization curves and (B) overpotential at 10 mA cm^{-2}. Note the MoZn ratio is only the precursors added in, not the true composition as Zn is more volatile than Mo.

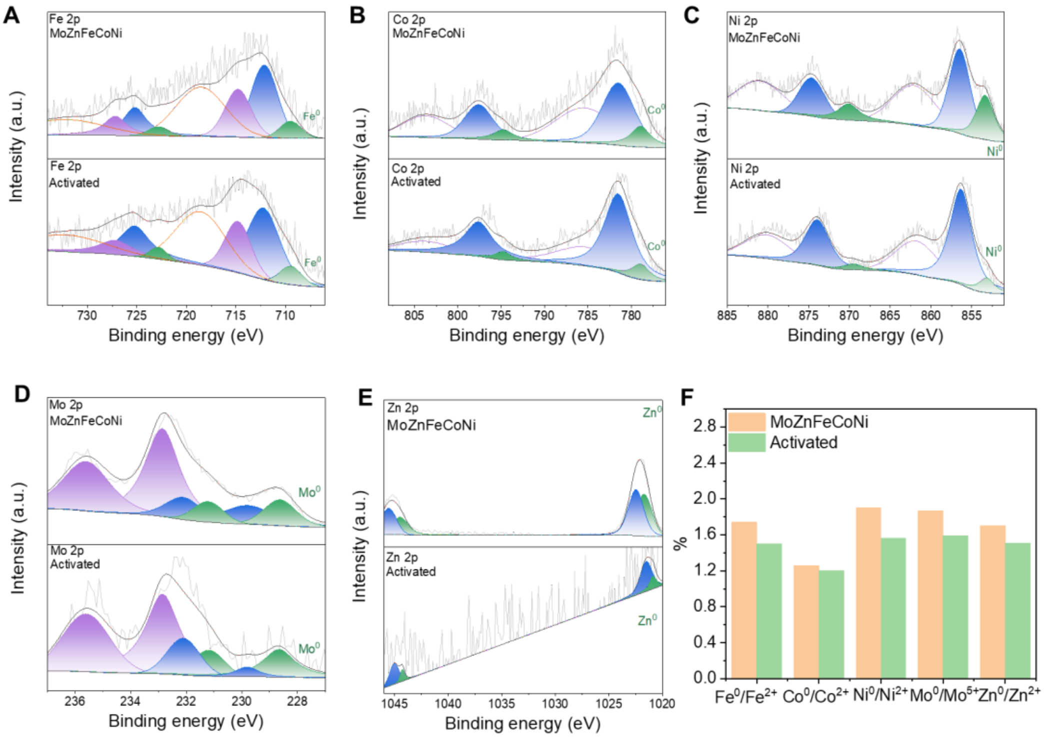

Fig. S9. Elemental valence state analyses. High-resolution XPS spectra of (A) Fe 2p, (B) Co 2p, (C) Ni 2p, (D) Mo 2p, and (E) Zn for the MoZn-HEA before and after electrocatalytic OER. (F) Changes in element oxidation states.

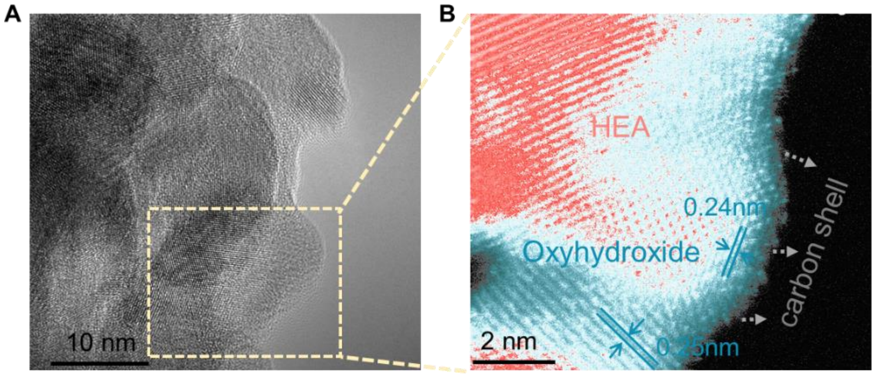

Fig. S10. TEM characterizations. (A)The HRTEM image of MoZnFeCoNi after anodic oxidation. (B) IFFT images obtained by selecting the blue- and red-color reflection spots in the FFT (from the yellow-cube region in (A)) showing that MOOH (blue-color) grows on the HEA matrix.

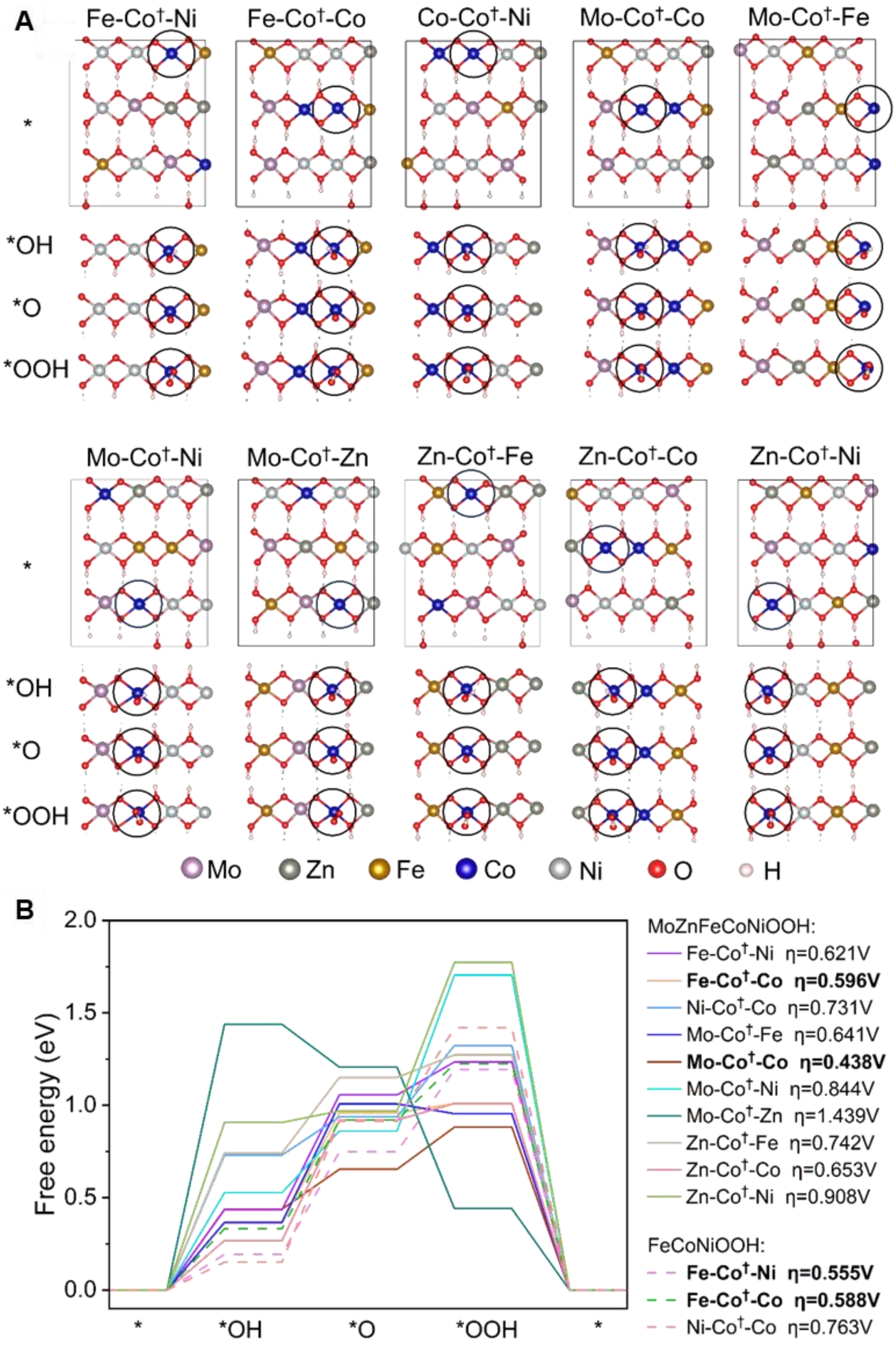

Fig. S11. AEM pathways and free energy diagrams. (A) The slab models and intermediate adsorption configurations along the AEM pathway for 10 different combinations of M_1-$Co^{\dagger}$-M_3 sites. The symbol "†" denotes the central Co atom as the active site, while M_1 and M_3 denote the neighboring metal atoms. For clarity, only the surface layer is shown; for the intermediate adsorption configurations, only the sites proximate to the active sites are shown. (B) The Gibbs free energies diagrams and corresponding AEM overpotentials (η) for OER steps at the M_1-$Co^{\dagger}$-M_3 sites in the MoZnFeCoNiOOH and FeCoNiOOH.

Site screening process for AEM pathways：

For the AEM pathway, metal sites are the active centers, denoted as M_1-$M_2^{\dagger}$-M_3 (**Fig. 5A**). Based on the partial DOS results in **Fig. 5B** and d-band centers listed in **Table S7**, Co atoms exhibit the highest lower Hubbard band center (LHB), and the states near the Fermi level are predominantly contributed by Co 3d-electrons, signifying Co as the primary active site. Hence, we focus on the Co atom as the active center (M_2 = Co) and explore the impact of neighboring atoms (M_1, M_3 = Mo, Zn, Fe, Co and Ni) on its OER performance.

In the case of M_1-$Co^{\dagger}$-M_3 sites in MoZnFeCoNiOOH, we investigated $C_5^2 = 10$ scenarios, each involving a pair from the five elements (pairs where M_1 and M_3 are identical are not considered here). These scenarios can be categorized into two groups:

Fe-$Co^{\dagger}$-Ni, Fe-$Co^{\dagger}$-Co, and Co-$Co^{\dagger}$-Ni, which are also present in FeCoNiOOH; Mo-$Co^{\dagger}$-Fe, Mo-$Co^{\dagger}$-Co, Mo-$Co^{\dagger}$-Ni, Mo-$Co^{\dagger}$-Zn, Zn-$Co^{\dagger}$-Fe, Zn-$Co^{\dagger}$-Co, and Zn-$Co^{\dagger}$-Ni, arising from the addition of Mo and Zn.

We randomly sample these combinations from a collection of randomly occupied MoZnFeCoNiOOH samples and investigated their OER performances along AEM pathway. **Fig. S11A** shows the slab models and intermediate adsorption configurations (*O, *OH and *OOH) for each combination. Feasible adsorption configurations can be achieved for each site (a slightly different scenario is for the Mo-$Co^{\dagger}$-Zn site, where the non-bonding states associated with Zn-coordinated O atoms lead to spontaneous decomposition of *OOH to *OO and *H upon optimization). **Fig. S11B** outlines the Gibbs free energies diagrams for these sites. The sites with overpotential (η) less than 0.6V are marked, which contribute to the AEM mechanism more notably. When compared to FeCoNiOOH, the overpotential remains nearly unchanged at the Fe-$Co^{\dagger}$-Co site, while being increased at the Fe-$Co^{\dagger}$-Ni site and decreased at the Ni-$Co^{\dagger}$-Co site, and changes in the AEM performance are not pronounced. The new combinations involving Mo and Zn, which are absent in FeCoNiOOH, show distinct behaviors. Among these, the Mo-$Co^{\dagger}$-Co site displays a notably lower overpotential of 0.438V, effectively enhancing the AEM pathway, while the Mo-$Co^{\dagger}$-Fe and Zn-$Co^{\dagger}$-Co sites also contribute with overpotentials around 0.65V. Although our investigation covered a limited number of sites, it is evident that the inclusion of Mo and Zn enhances surface complexity and leads to a more continuous distribution of adsorption energies, increasing the likelihood of finding optimal active sites.

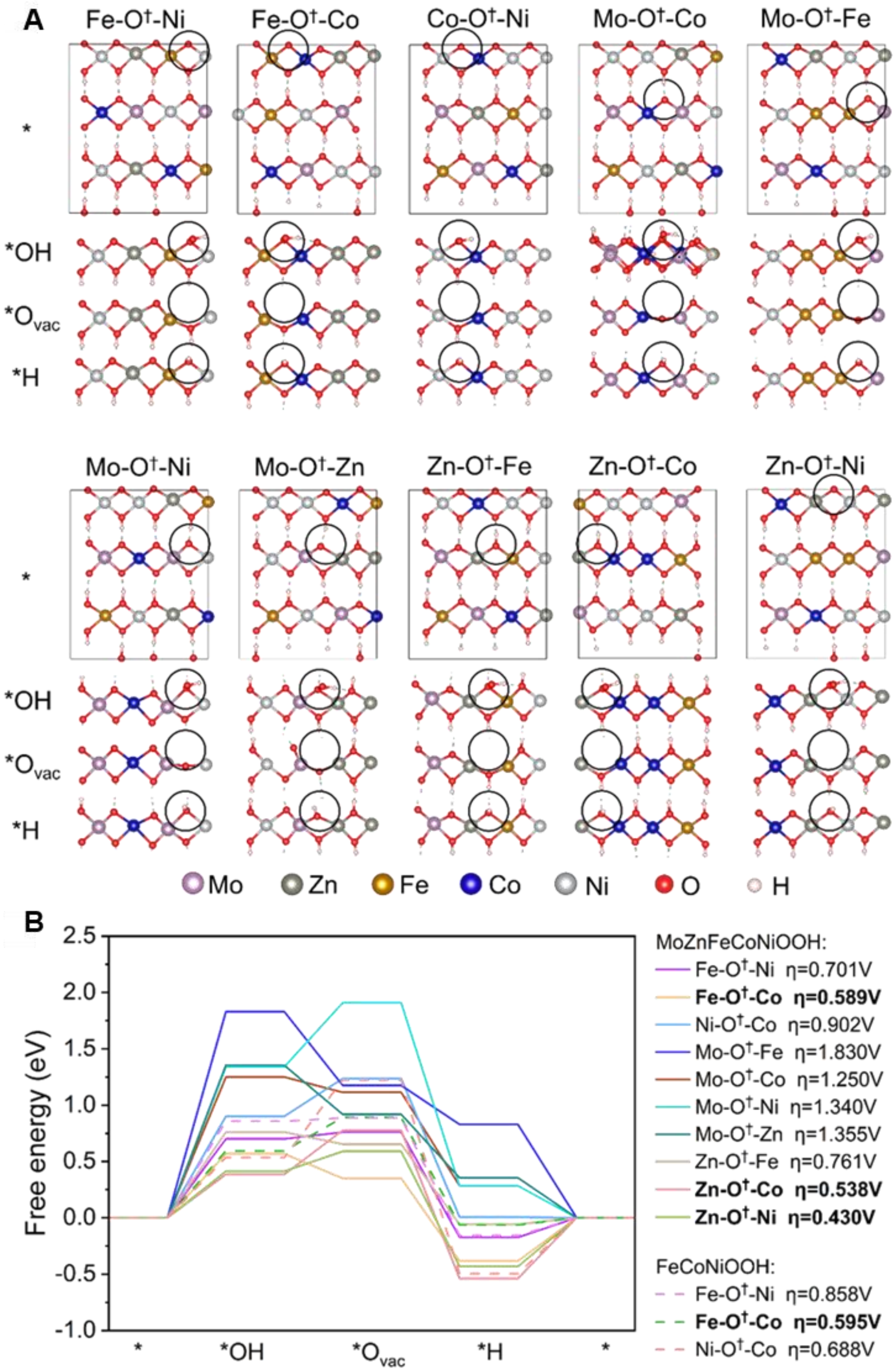

Fig. S12. LOM pathways and free energy diagrams. (A) The slab models and intermediate adsorption configurations along the LOM pathway for 10 different combinations of M_1-O†-M_2 sites. The symbol "†" denotes the central O atom as the active site, while M_1 and M_2 denote the neighboring metal atoms. For clarity, only the surface layer is shown; for the intermediate adsorption configurations, only the sites proximate to the active sites are shown. (B) The Gibbs free energies diagrams and corresponding LOM overpotentials (η) for OER steps at the M_1-O†-M_2 sites in the MoZnFeCoNiOOH and FeCoNiOOH.

Site screening process for LOM pathways：

For LOM, the O atoms between different metals serve as the active centers, denoted as M_1-O†-M_2. We also investigated $C_5^2 = 10$ scenarios to explore the neighboring effects:

Fe-O†-Ni, Fe-O†-Co, and Co-O†-Ni, common in FeCoNiOOH. Mo-O†-Fe, Mo-O†-Co, Mo-O†-Ni, Mo-O†-Zn, Zn-Co†-Fe, Zn-Co†-Co, and Zn-Co†-Ni, stemming from the inclusion of Mo and Zn.

The results for these M_1-O†-M_2 combinations are presented in **Fig. S12**. Similar to FeCoNiOOH, the Fe-O†-Co site also exhibits a relatively low overpotential of 0.589V. However, the new inclusion of Zn introduces the Zn-Co†-Ni and Zn-Co†-Co sites, which show more optimal overpotentials of 0.430V and 0.538V, respectively. As discussed in the manuscript (**Fig. 5B**), these improvements are attributed to the upshift of the O-2p band center and the weakening of Zn-O bonds, which enhances the activity of lattice oxygen and facilitate the occurrence of LOM processes.

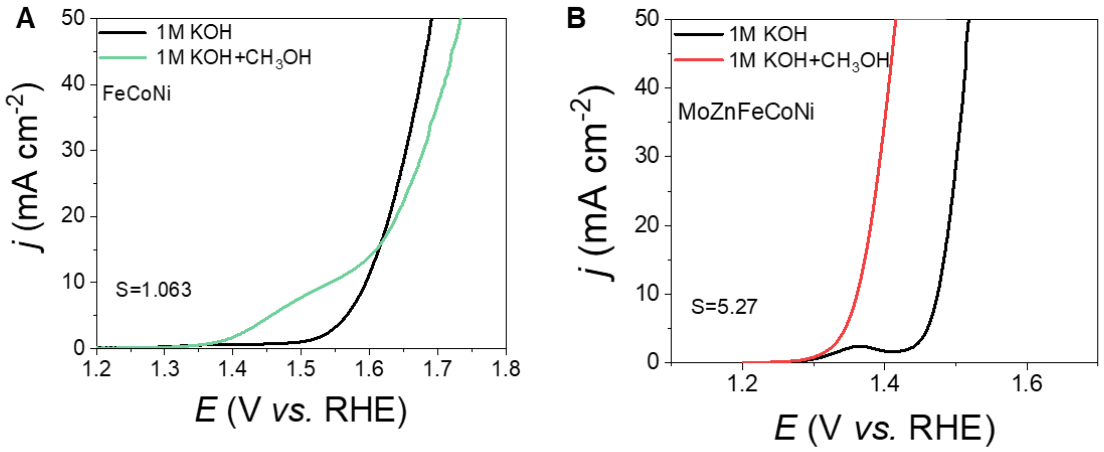

Fig. S13. Polarization curves. LSV curves of (A–B) FeCoNi and MoZnFeCoNi in 1.0 M KOH (with and without methanol) (0.602 mol L^{-1}).

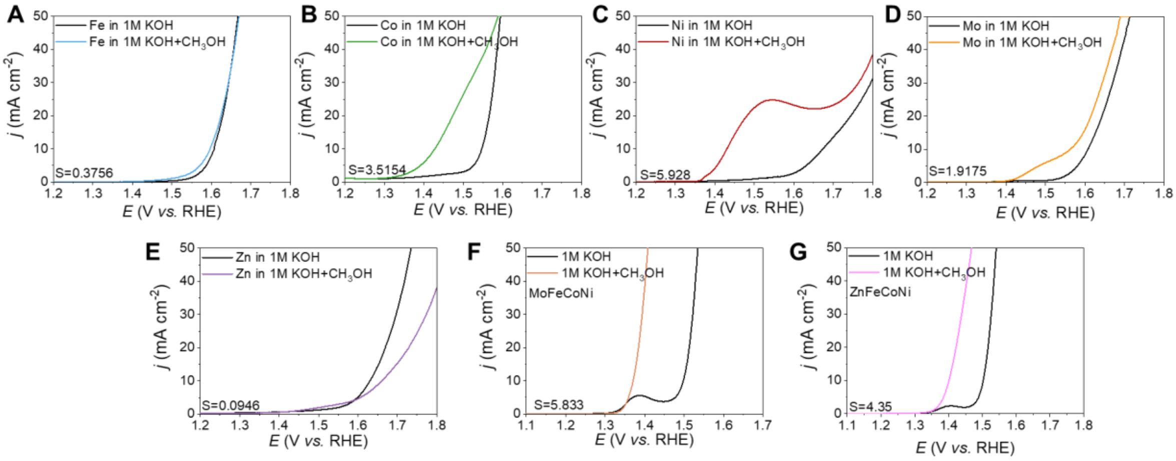

Fig. S14. **Polarization curves.** LSV curves of (A) Fe, (B) Co, (C)Ni, (D)Mo, (E)Zn, (F) MoFeCoNi and (G) ZnFeCoNi in 1.0 M KOH (with and without methanol) (0.602 mol L^{-1}).

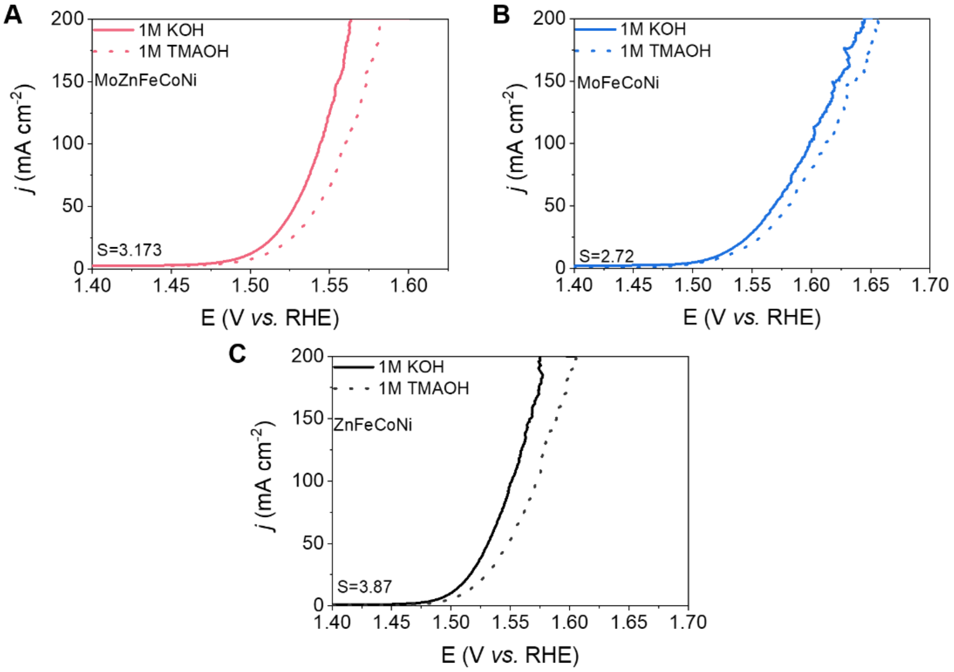

Fig. S15. Polarization curves. (A) MoZnFeCoNi, (B) MoFeCoNi and (C) ZnFeCoNi in 1.0 M KOH and TMAOH.

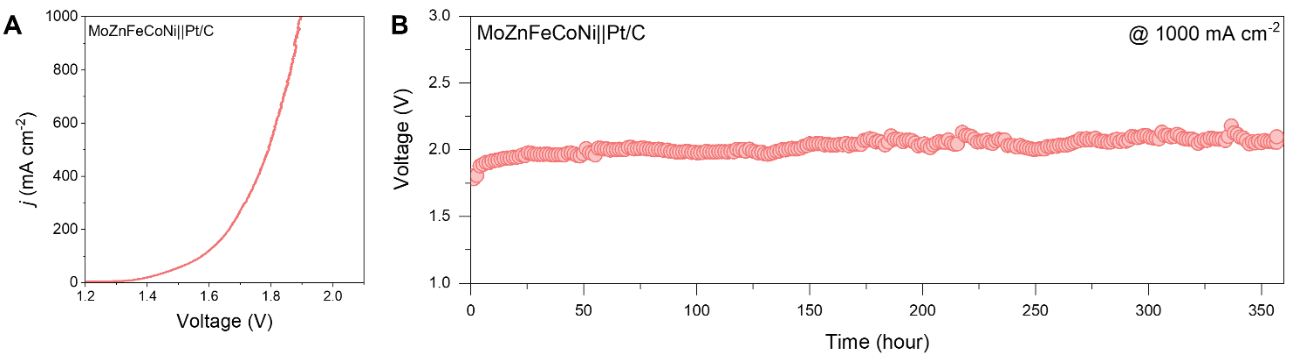

Fig. S16. Stability test and polarization curves and comparison of stability. (A) LSV polarization curves of MoZn-HEA||Pt/C for the alkaline water splitting. (B) Chronopotentiometry tests of MoZn-HEA||Pt/C in 1.0 M KOH at 1000 mA cm^{-2}.

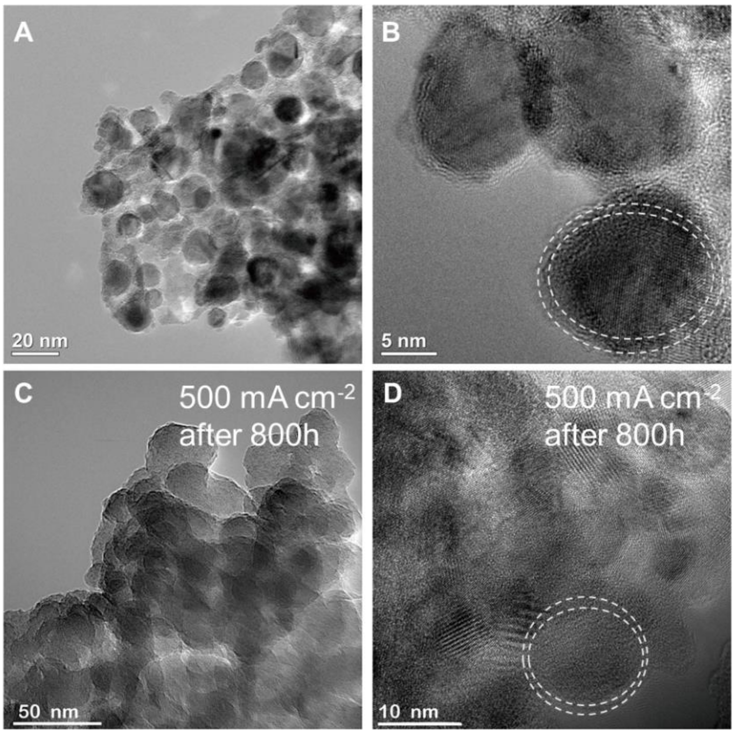

Fig. S17. TEM characterizations. The HRTEM of MoZnFeCoNi (A-B) before and (C-D) after electrocatalytic OER stability.

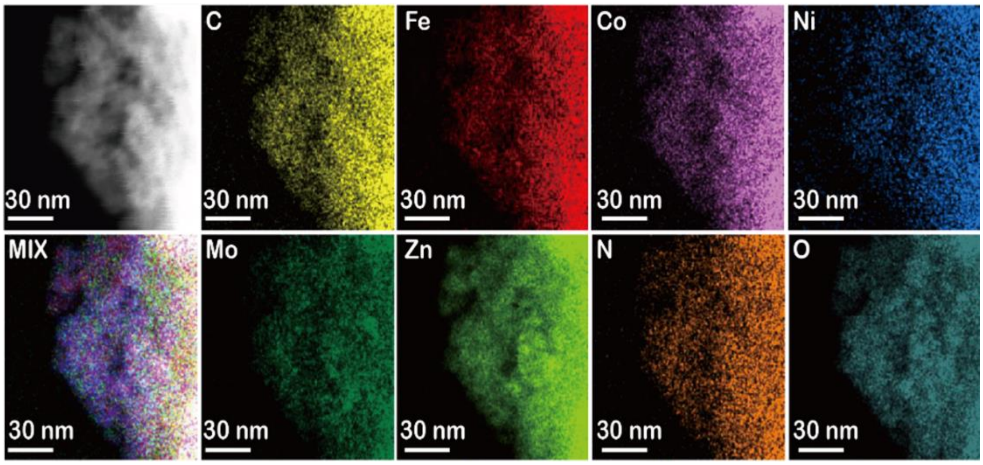

Fig. S18. TEM characterizations. Electrocatalytic OER stability and structure characterizations of the prepared MoZnFeCoNi in 1.0 M KOH at 500 mA cm^{-2}

Supporting Tables

Table S1. Compositional analyses. The Zn content of catalysts measured by XPS and ICP under different defined synthesis conditions.

Catalyst	Zn (XPS)	Zn (ICP)
ST-HEA	5.75	6.06
S-HEA	0.00	0.89
T-HEA	1.05	1.9

Table S2. Comparison of specific surface area. The BET of various reported HEA catalysts.

Sample	S_{BET} (m^2 g^{-1})	method	Ref.
ST-HEA	139.25	Spatiotemporal-confined engineering (0.1 s)	This work
MOF-74-H-650	120	Pyrolysis in tube furnace (3 h)	(*54*)
np-NCMCF HEA	16.42	Dealloying (4 h)	(*55*)
CuCrFeNiCoP	54.6	Electrodeposition (40 min)	(29)
AlNiCuCoFeY (HEF/Al alloy)	16.15	Fluorination (2 h)	(*56*)
HEACB 1000	84	Annealing (2 h)	(*57*)
FeCoNiRu-450	44.8	Carbonization (2 h)	(*10*)
$NiFeXO_4$ (X = Fe, Ni, Al, Mo, Co, Cr)	137.0	Immersing (8 h)	(*4*)

Table S3. Compositional analyses. The element content of catalysts measured by ICP-OER

Catalyst	Zn (%)	Mo (%)	Fe (%)	Co (%)	Ni (%)
ST-HEA	6.07	24.75	26.91	12.58	29.64
S-HEA	0.89	19.01	16.88	22.98	40.24
T-HEA	1.92	23.2	20.09	19.52	35.27
HEM (High entropy MOF)	9.59	36.76	25.11	9.59	18.95
ST-HEA activated	3.56	25.83	27.25	12.04	31.32
ST-HEA after stability	1.34	10.14	34.65	19.34	34.53

Table S4. Comparisons of performance. The alkaline OER performance of recently reported most active high-entropy catalysts.

Number	Catalyst	Electrolyte	Overpotential	Tafel slope (mV dec^{-1})	Stability	Ref.
**1**	**MoZnFeCoNi**	**1.0 M KOH**	**220 mV @ 10 mA cm^{-2}**	**48.78**	**1600 @ 100mA cm^{-2}**	**This work**
2	Co-Fe-Ga-Ni-Zn	1.0 M KOH	370 mV @ 10 mA cm^{-2}	71	10h @ 10mA cm^{-2}	(*21*)
3	MnFeCoNi HEA	1.0 M KOH	302 mV @ 10 mA cm^{-2}	83.7	20h @ 10mA cm^{-2}	(*25*)
4	CoCrFeMnNiP HEMP	1.0 M KOH	320 mV @ 10 mA cm^{-2}	60.8	24h @ 10mA cm^{-2}	(*26*)
5	CoCrFeNiMo-20 Mg HEA	1.0 M KOH	220 mV @ 10 mA cm^{-2}	59	24h @ 100mA cm^{-2}	(*27*)
6	Fe-Co-Ni-Cr-Nb MCPS	1.0 M KOH	288 mV @10 mA cm^{-2}	27.7	30h @ 10mA cm^{-2}	(*28*)
7	CoCrFeNiAl HEC	1.0 M KOH	240 mV @10 mA cm^{-2}	---	240h @ 10mA cm^{-2}	(*58*)
8	$Mg_{0.2}Co_{0.2}Ni_{0.2}Cu_{0.2}Zn_{0.2}O$ HEO	1.0 M KOH	360 mV @10 mA cm^{-2}	61.4	25h @ 10mA cm^{-2}	(*30*)
9	MnFeCoNiCu HE-MOF-ST	1.0 M KOH	293 mV @10 mA cm^{-2}	81	48h @ 10mA cm^{-2}	(*31*)
10	FeNiMnCrCu HEA	1.0 M KOH	317mV @10 mA cm^{-2}	58	10h @26 mA cm^{-2}	(*32*)
11	FeCoNiCrAl HEA	1.0 M KOH	342 mV @10 mA cm^{-2}	75	---	
12	AlNiFeCoMo HEO	1.0 M KOH	240 mV @10 mA cm^{-2}	46	50h @ 20 mA cm^{-2}	(*33*)
13	AlCrCuFeNi HEAs	1.0 M KOH	270 mV @10 mA cm^{-2}	77.5	35h @ 17.5 mA cm^{-2}	(*34*)
14	$Fe_{29}Co_{27}Ni_{23}Si_{9}B_{12}$-HEA-3h	1.0 M KOH	277 mV @10 mA cm^{-2}	85	50h @ 100 mA cm^{-2}	(*59*)
15	$(Fe_{0.73}Cr_{0.71}Co_{0.78}Ni_{0.81}Al_{0.1})O_{4.01}$ HEO	1.0 M KOH	381 mV @10 mA cm^{-2}	97.4	120h @ 10 mA cm^{-2}	(*60*)
16	HF-CoCrFeNiAl HEA	1.0 M KOH	265 mV @10 mA cm^{-2}	56.8	10h @ 10 mA cm^{-2}	(*61*)
17	Fe-Cr-Co-Ni-Cu HE-LDHs-Ar-20	1.0 M KOH	330mV @ 10 mA cm^{-2}	63.7	24h @ 10 mA cm^{-2}	(*62*)
18	MnFeCoNiCu	1.0 M KOH	263 mV @ 10 mA cm^{-2}	43	24h @ 10 mA cm^{-2}	(*63*)
19	FeCoNiMo HEA	1.0 M KOH	250 mV @ 10 mA cm^{-2}	42.5	65h @ 10 mA cm^{-2}	(*6*)
20	$Pt_{34}Fe_{5}Ni_{20}Cu_{31}Mo_{9}Ru$ HEA	1.0 M KOH	259 mV @ 10 mA cm^{-2}	39	40h @ 10 mA cm^{-2}	(*64*)
21	FeCoNiMoW HEA	1.0 M KOH	233 mV/276 mV @ 10 /100 mA cm^{-2}	36.7	40 @ 10 mA cm^{-2}	(*3*)
22	CrMnFeCoNi	1.0 M KOH	265 mV @ 10 mA cm^{-2}	37.9	30h @ 10 mA cm^{-2}	(*35*)
23	FeCoNiCrMo	1.0 M KOH	303 mV @ 100 mA cm^{-2}	38.5	120 @ 50 mA cm^{-2}	(*36*)
24	FeCoNiRu-450	1.0 M KOH	243 mV @ 10 mA cm^{-2}	45	40 @ 10 mA cm^{-2}	(*10*)
25	np-NiFeCoMnOOH	1.0 M KOH	194 mV @ 10 mA cm^{-2}	67.96	120h @ 100 mA cm^{-2}	(*65*)
26	CNF@FeNiCo	1.0 M KOH	445.6mV @ 10 mA cm^{-2}	71.5	/	(*66*)
27	$NiFeXO_4$ (X = FeNiAlMoCoCr)	1.0 M KOH	195 mV @ 10 mA cm^{-2}	53.3	120h @ 10 mA cm^{-2}	(*4*)

Table S5. Surface oxygen compositions. Proportions of M−O, M−OH and M−OOH in the before OER and after activated HEA catalysts measured by the corresponding XPS fitting data.

Catalysts	M−O (%)	M−OH (%)	M−OOH (%)
Before OER	--	44.88	55.12
After activated	9.7	47.98	42.92

Table S6. Compositional analyses. The metal content of catalysts measured by XPS on catalysts surface structure.

Catalyst	Zn (%)	Mo (%)	Fe (%)	Co (%)	Ni (%)
ST-HEA	5.70	22.00	19.86	24.38	28.06
ST-HEA activated	3.10	20.29	18.62	24.68	33.32

Table S7. The centers of LHB and UHB of characterization. The centers of the filled lower Hubbard band (LHB) and the empty upper Hubbard band (UHB) of the MoZnFeCoNiOOH and FeCoNiOOH surface atoms as well as of each element on the surface.

	MoZnFeCoNiOOH		FeCoNiOOH	
	**LHB center**	**UHB center**	**LHB center**	**UHB center**
Overall	-4.794	2.693	-4.342	2.305
Fe	-5.880	2.422	-5.790	2.276
Co	-3.416	2.156	-3.206	2.417
Ni	-4.181	2.407	-4.302	2.201
Mo	-5.383	3.181		
Zn	-5.451	/		

Table S8. Comparisons of stability. Comparisons of the OER stability and multistage structure synthesis time for electrocatalysts prepared by different strategies.

Electrocatalysts	Preparation methods	Reaction time	OER stability	Ref
**MoZnFeCoNi**	**Spatiotemporal-confined engineering**	**0.1 s**	**1546 h@ 100 mA cm^{-2}**<br>**897.6 h@500 mA cm^{-2}**	**This work**
ZIF-67@$Co(OH)_2$	impregnation method	12 h	24 h @ 500 mA cm^{-2}	(*67*)
np-Co@CoO/RuO_2	vapor phase dealloying	72 h	50 h @ 500 mA cm^{-2}	(*68*)
M-NiA-CoN	mechanochemical post-synthesis	1 h	65 h @ 10 mA cm^{-2}	(*69*)
core–shell FeCoNiMoAl-based HEA	dealloying	4 h	330 h @ 2 mA cm^{-2}	(*70*)
NLOS-1	pyrolysis on tube furnace	2 h	72 h @ 100 mA cm^{-2}	(*71*)
Ce-CoP	phosphorization	2 h	27 h @ 10 mA cm^{-2}	(*72*)

Table S9. Compositional analyses. Dissolved amount of each metal in the catalysts find in the electrolyte solution after OER long-term tests, as measured by ICP-MS.

Current density (mA cm^{-2})	Catalysts	KOH (mg/L)	Fe (mg/L)	Co (mg/L)	Ni (mg/L)	Mo (mg/L)	Zn (mg/L)	
**100**	***MoZn-HEA***	**59000**	**0.039**	**0.038**	**0**	**0.172**	**1.905**	**This work**
**500**	***MoZn-HEA***	**59000**	**1.9**	**0.79**	**3.88**	**3.8**	**2.34**	
100	ZnFeCoNi	59000	0.1642	0.1507	0.2939	0	2.31	
100	FeCoNiAlCe HEO	/	0.15	0.04	0.08	/	/	**(*73*)**

REFERENCES AND NOTES

1. Z. F. Huang, J. J. Song, Y. H. Du, S. B. Xi, S. Dou, J. M. V. Nsanzimana, C. Wang, Z. C. J. Xu, X. Wang, Chemical and structural origin of lattice oxygen oxidation in Co-Zn oxyhydroxide oxygen evolution electrocatalysts. *Nat. Energy* **4**, 329–338 (2019).

2. H. B. Tao, Y. H. Xu, X. Huang, J. Z. Chen, L. J. Pei, J. M. Zhang, J. G. G. Chen, B. Liu, A general method to probe oxygen evolution intermediates at operating conditions. *Joule* **3**, 1498–1509 (2019).

3. R. He, L. L. Yang, Y. Zhang, D. Jiang, S. Lee, S. Horta, Z. F. Liang, X. Lu, A. O. Moghaddam, J. S. Li, M. Ibáñez, Y. Xu, Y. T. Zhou, A. Cabot, A 3d-4d-5d high entropy alloy as a bifunctional oxygen catalyst for robust aqueous zinc-air batteries. *Adv. Mater.* **35**, 202303719 (2023).

4. Q. W. Zhang, Y. X. Hu, H. F. Wu, X. R. Zhao, M. L. Wang, S. H. Wang, R. H. Feng, Q. Chen, F. Song, M. W. Chen, P. Liu, Entropy-stabilized multicomponent porous spinel nanowires of $NiFe_xO_4$ (x = Fe, Ni, Al, Mo, Co, Cr) for efficient and durable electrocatalytic oxygen evolution reaction in alkaline medium. *ACS Nano* **17**, 1485–1494 (2023).

5. X. Wang, H. Zhong, S. Xi, W. S. V. Lee, J. Xue, Understanding of oxygen redox in the oxygen evolution reaction. *Adv. Mater.* **34**, 202107956 (2022).

6. Y. Mei, Y. Feng, C. Zhang, Y. Zhang, Q. Qi, J. Hu, High-entropy alloy with Mo-coordination as efficient electrocatalyst for oxygen evolution reaction. *ACS Catal.* **12**, 10808–10817 (2022).

7. M. Lu, Y. Zheng, Y. Hu, B. L. Huang, D. G. Ji, M. Z. Sun, J. Y. Li, Y. Peng, R. Si, P. X. Xi, C. H. Yan, Artificially steering electrocatalytic oxygen evolution reaction mechanism by regulating oxygen defect contents in perovskites. *Sci. Adv.* **8**, eabq3563 (2022).

8. Z. Y. He, J. Zhang, Z. H. Gong, H. Lei, D. Zhou, N. A. Zhang, W. J. Mai, S. J. Zhao, Y. Chen, Activating lattice oxygen in NiFe-based (Oxy)hydroxide for water electrolysis. *Nat. Commun.* **13**, 2191 (2022).

9. A. K. Tomar, U. N. Pan, N. H. Kim, J. H. Lee, Enabling lattice oxygen participation in a triple perovskite oxide electrocatalyst for the oxygen evolution reaction. *ACS Energy Lett.* **8**, 565–573 (2022).

10. K. Huang, J. Y. Xia, Y. Lu, B. W. Zhang, W. C. Shi, X. Cao, X. Y. Zhang, L. M. Woods, C. C. Han, C. J. Chen, T. Wang, J. S. Wu, Y. Z. Huang, Self-reconstructed spinel surface structure enabling the long-term stable hydrogen evolution reaction/oxygen evolution reaction efficiency of feconiru high-entropy alloyed electrocatalyst. *Adv. Sci.* **10**, 202300094 (2023).

11. Y. G. Yao, Z. N. Huang, P. F. Xie, S. D. Lacey, R. J. Jacob, H. Xie, F. J. Chen, A. M. Nie, T. C. Pu, M. Rehwoldt, D. W. Yu, M. R. Zachariah, C. Wang, R. Shahbazian-Yassar, J. Li, L. B. Hu, Carbothermal shock synthesis of high-entropy-alloy nanoparticles. *Science* **359**, 1489–1494 (2018).

12. Z. Jing, Y. Guo, Q. Wang, X. Yan, G. Yue, Z. Li, H. Liu, R. Qin, C. Zhong, M. Li, D. Xu, Y. Yao, Y. Yao, M. Shuai, Ambient hydrogenation of solid aromatics enabled by a high entropy alloy nanocatalyst. *Nat. Commun.* **15**, 5806 (2024).

13. L. L. Yu, K. Z. Zeng, C. H. Li, X. R. Lin, H. W. Liu, W. H. Shi, H. J. Qiu, Y. F. Yuan, Y. G. Yao, High-entropy alloy catalysts: From bulk to nano toward highly efficient carbon and nitrogen catalysis. *Carbon Energy* **4**, 731–761 (2022).

14. L. He, M. Li, L. Qiu, S. Geng, Y. Liu, F. Tian, M. Luo, H. Liu, Y. Yu, W. Yang, S. Guo, Single-atom Mo-tailored high-entropy-alloy ultrathin nanosheets with intrinsic tensile strain enhance electrocatalysis. *Nat. Commun.* **15**, 2290–2290 (2024).

15. Z. J. Li, S. Q. Ji, C. Wang, H. X. Liu, L. P. Leng, L. Du, J. C. Gao, M. Qiao, J. H. Horton, Y. Wang, Geometric and electronic engineering of atomically dispersed copper-cobalt diatomic sites for synergistic promotion of bifunctional oxygen electrocatalysis in zinc-air batteries. *Adv. Mater.* **35**, 202300905 (2023).

16. M. Miao, J. Pan, T. He, Y. Yan, B. Y. Xia, X. Wang, Molybdenum carbide-based electrocatalysts for hydrogen evolution reaction. *Chem. Eur. J.* **23**, 10947–10961 (2017).

17. Y. G. Yao, Z. N. Huang, L. A. Hughes, J. L. Gao, T. Y. Li, D. Morris, S. E. Zeltmann, B. H. Savitzky, C. Ophus, Y. Z. Finfrock, Q. Dong, M. L. Jiao, Y. M. Mao, M. F. Chi, P. Zhang, J. Li, A. M. Minor, R. Shahbazian-Yassar, L. B. Hu, Extreme mixing in nanoscale transition metal alloys. *Matter* **4**, 2340–2353 (2021).

18. W. H. Shi, Z. Z. Li, Z. H. Gong, Z. H. Liang, H. W. Liu, Y. C. Han, H. T. Niu, B. Song, X. D. Chi, J. H. Zhou, H. Wang, B. Y. Xia, Y. G. Yao, Z. Q. Tian, Transient and general synthesis of high-density and ultrasmall nanoparticles on two-dimensional porous carbon via coordinated carbothermal shock. *Nat. Commun.* **14**, 2294 (2023).

19. Y. X. Wang, Y. Zhang, P. Y. Xing, X. Q. Li, Q. Y. Du, X. Q. Fan, Z. B. Cai, R. Yin, Y. G. Yao, W. T. Gan, Self-encapsulation of high-entropy alloy nanoparticles inside carbonized wood for highly durable electrocatalysis. *Adv. Mater.* **36**, 02391 (2024).

20. B. Talluri, K. Yoo, J. Kim, High entropy spinel metal oxide $(CoCrFeMnNi)_3O_4$ nanoparticles as novel efficient electrocatalyst for methanol oxidation and oxygen evolution reactions. *J. Environ. Chem. Eng.* **10**, 106932 (2022).

21. L. Sharma, N. K. Katiyar, A. Parui, R. Das, R. Kumar, C. S. Tiwary, A. K. Singh, A. Halder, K. Biswas, Low-cost high entropy alloy (HEA) for high-efficiency oxygen evolution reaction (OER). *Nano Res.* **15**, 4799–4806 (2022).

22. P. Li, X. Wan, J. Su, W. Liu, Y. Guo, H. Yin, D. Wang, A single-phase FeCoNiMnMo high-entropy alloy oxygen evolution anode working in alkaline solution for over 1000 h. *ACS Catal.* **11667**, 11667–11674 (2022).

23. L. He, N. Wang, B. Sun, L. Zhong, M. Yao, W. Hu, S. Komarneni, High-entropy FeCoNiMn (Oxy)hydroxide as high-performance electrocatalyst for OER and boosting clean carrier production under quasi-industrial condition. *J. Clean. Prod.* **356**, 131680 (2022).

24. Z. Jin, J. Lyu, Y.-L. Zhao, H. Li, X. Lin, G. Xie, X. Liu, J.-J. Kai, H.-J. Qiu, Rugged high-entropy alloy nanowires with in situ formed surface spinel oxide as highly stable electrocatalyst in zn–air batteries. *ACS Mater. Lett.* **2**, 1698–1706 (2020).

25. W. Dai, T. Lu, Y. Pan, Novel and promising electrocatalyst for oxygen evolution reaction based on MnFeCoNi high entropy alloy. *J. Power Sources* **430**, 104–111 (2019).

26. X. H. Zhao, Z. M. Xue, W. J. Chen, Y. Q. Wang, T. C. Mu, Eutectic synthesis of high-entropy metal phosphides for electrocatalytic water splitting. *Chemsuschem* **13**, 2038–2042 (2020).

27. J. Tang, J. L. Xu, Z. G. Ye, X. B. Li, J. M. Luo, Microwave sintered porous CoCrFeNiMo high entropy alloy as an efficient electrocatalyst for alkaline oxygen evolution reaction. *J. Mater. Sci. Technol.* **79**, 171–177 (2021).

28. Z. Ding, J. Bian, S. Shuang, X. Liu, Y. Hu, C. Sun, Y. Yang, High entropy intermetallic-oxide core-shell nanostructure as superb oxygen evolution reaction catalyst. *Adv. Sustain. Syst.* **4**, 1900105 (2020).

29. T. Zhang, J. Li, B. Zhang, G. Wang, K. Jiang, Z. Zheng, J. Shen, High-entropy alloy CuCrFeNiCoP film of Cu-based as high-efficiency electrocatalyst for water splitting. *J. Alloys Compd.* **969**, 172439 (2023).

30. F. M. Liu, M. Yu, X. Chen, J. H. Li, H. H. Liu, F. Y. Cheng, Defective high-entropy rocksalt oxide with enhanced metal-oxygen covalency for electrocatalytic oxygen evolution. *Chinese J. Catal.* **43**, 122–129 (2022).

31. X. H. Zhao, Z. M. Xue, W. J. Chen, X. Y. Bai, R. F. Shi, T. C. Mu, Ambient fast, large-scale synthesis of entropy-stabilized metal-organic framework nanosheets for electrocatalytic oxygen evolution. *J. Mater. Chem. A* **7**, 26238–26242 (2019).

32. X. D. Cui, B. L. Zhang, C. Y. Zeng, S. M. Guo, Electrocatalytic activity of high-entropy alloys toward oxygen evolution reaction. *MRS Commun.* **8**, 1230–1235 (2018).

33. H. J. Qiu, G. Fang, J. J. Gao, Y. R. Wen, J. Lv, H. L. Li, G. Q. Xie, X. J. Liu, S. H. Sun, Noble metal-free nanoporous high-entropy alloys as highly efficient electrocatalysts for oxygen evolution reaction. *ACS Mater. Lett.* **1**, 526–533 (2019).

34. L. H. Liu, N. Li, M. Han, J. R. Han, H. Y. Liang, Scalable synthesis of nanoporous high entropy alloys for electrocatalytic oxygen evolution. *Rare Met.* **41**, 125–131 (2022).

35. R. He, L. L. Yang, Y. Zhang, X. Wang, S. Lee, T. Zhang, L. X. Li, Z. F. Liang, J. W. Chen, J. S. Li, A. O. Moghaddam, J. Llorca, M. Ib, J. Arbiol, Y. Xu, A. Cabot, A CrMnFeCoNi high entropy alloy boosting oxygen evolution/reduction reactions and zinc-air battery performance. *Energy Storage Mater.* **58**, 287–298 (2023).

36. L. Y. Yi, S. M. Xiao, Y. P. Wei, D. Z. Li, R. F. Wang, S. F. Guo, W. H. Hu, Free-standing high-entropy alloy plate for efficient water oxidation catalysis: Structure/composition evolution and implication of high-valence metals. *Chem. Eng. J.* **469**, 144015 (2023).

37. R. R. Zhang, L. Pan, B. B. Guo, Z. F. Huang, Z. X. Chen, L. Wang, X. W. Zhang, Z. Y. Guo, W. Xu, K. P. Loh, J. J. Zou, Tracking the role of defect types in Co_3O_4 structural evolution and active motifs during oxygen evolution reaction. *J. Am. Chem. Soc.* **145**, 2271–2281 (2023).

38. C. J. Hu, Y. F. Hu, C. H. Fan, L. Yang, Y. T. Zhang, H. X. Li, W. Xie, Surface-enhanced raman spectroscopic evidence of key intermediate species and role of NiFe dual-catalytic center in water oxidation. *Angew. Chem. Int. Ed.* **60**, 19774–19778 (2021).

39. C. Wang, P. L. Zhai, M. Y. Xia, W. Liu, J. F. Gao, L. C. Sun, J. A. Hou, Identification of the origin for reconstructed active sites on oxyhydroxide for oxygen evolution reaction. *Adv. Mater.* **35**, 09307 (2023).

40. H. Q. Chu, R. J. Li, P. P. Feng, D. Y. Wang, C. X. Li, Y. L. Yu, M. Yang, Ligands defect-induced structural self-reconstruction of Fe-Ni-Co-hydroxyl oxides with crystalline/amorphous heterophase from a 2D metal-organic framework for an efficient oxygen evolution reaction. *ACS Catal.* **14**, 1553–1566 (2024).

41. E. Fabbri, M. Nachtegaal, T. Binninger, X. Cheng, B. J. Kim, J. Durst, F. Bozza, T. Graule, R. Schäublin, L. Wiles, M. Pertoso, N. Danilovic, K. E. Ayers, T. J. Schmidt, Dynamic surface self-reconstruction is the key of highly active perovskite nano-electrocatalysts for water splitting. *Nat. Mater.* **16**, 925–931 (2017).

42. L. Z. Wei, M. D. Hossain, M. J. Boyd, J. Aviles-Acosta, M. E. Kreider, A. C. Nielander, M. B. Stevens, T. F. Jaramillo, M. Bajdich, C. Hahn, Insights into active sites and mechanisms of

benzyl alcohol oxidation on nickel-iron oxyhydroxide electrodes. *ACS Catal.* **13**, 4272–4282 (2023).

43. Y. M. Sun, H. B. Liao, J. R. Wang, B. Chen, S. N. Sun, S. J. H. Ong, S. B. Xi, C. Z. Diao, Y. H. Du, J. O. Wang, M. B. H. Breese, S. Z. Li, H. Zhang, Z. C. J. Xu, Covalency competition dominates the water oxidation structure-activity relationship on spinel oxides. *Nat. Catal.* **3**, 959–959 (2020).

44. B. Zhang, L. Wang, Z. Cao, S. M. Kozlov, F. P. G. de Arquer, C. T. Dinh, J. Li, Z. Y. Wang, X. L. Zheng, L. S. Zhang, Y. Z. Wen, O. Voznyy, R. Comin, P. De Luna, T. Regier, W. L. Bi, E. E. Alp, C. W. Pao, L. R. Zheng, Y. F. Hu, Y. J. Ji, Y. Y. Li, Y. Zhang, L. Cavallo, H. S. Peng, E. H. Sargent, High-valence metals improve oxygen evolution reaction performance by modulating 3D metal oxidation cycle energetics. *Nat. Catal.* **3**, 985–992 (2020).

45. J. Wang, J. Zhang, L. Zhang, L. Chen, G. He, H. Jiang, Modulating metal-oxygen interactions of high-entropy oxide electrocatalysts enables highly-active and ultra-stable water oxidation. *Appl. Catal. B. Environ.* **342**, 123382 (2024).

46. J. P. Perdew, K. Burke, M. Ernzerhof, Generalized gradient approximation made simple. *Phys. Rev. Lett.* **77**, 3865–3868 (1996).

47. P. E. Blöchl, Projector augmented-wave method. *Phys. Rev. B* **50**, 17953–17979 (1994).

48. J. Baek, M. D. Hossain, P. Mukherjee, J. H. Lee, K. T. Winther, J. Leem, Y. Jiang, W. C. Chueh, M. Bajdich, X. L. Zheng, Synergistic effects of mixing and strain in high entropy spinel oxides for oxygen evolution reaction. *Nat. Commun.* **14**, 5936 (2023).

49. K. Winther, M. Hoffmann, O. Mamun, J. Boes, M. Bajdich, T. Bligaard, Catalysis-hub.Org: An open electronic structure database for surface reactions and catalytic materials. *Abstr. Pap. Am. Chem. Soc.* **257**, 88 (2019).

50. H. J. Monkhorst, J. D. Pack, Special points for brillouin-zone integrations. *Phys. Rev. B* **13**, 5188–5192 (1976).

51. S. Grimme, J. Antony, S. Ehrlich, H. Krieg, A consistent and accurate ab initio parametrization of density functional dispersion correction (DFT-D) for the 94 elements h-pu. *J. Chem. Phys.* **132**, 154104 (2010).

52. G. Henkelman, A. Arnaldsson, H. Jónsson, A fast and robust algorithm for bader decomposition of charge density. *Comput. Mater. Sci.* **36**, 354–360 (2006).

53. J. K. Nørskov, J. Rossmeisl, A. Logadottir, L. Lindqvist, J. R. Kitchin, T. Bligaard, H. Jónsson, Origin of the overpotential for oxygen reduction at a fuel-cell cathode. *J. Phys. Chem. B.* **108**, 17886–17892 (2004).

54. J. Hu, L. Cao, Z. Wang, J. Liu, J. Zhang, Y. Cao, Z. Lu, H. Cheng, Hollow high-entropy metal organic framework derived nanocomposite as efficient electrocatalyst for oxygen reduction reaction. *Compos. Commun.* **27**, 100866 (2021).

55. Z.-J. Zhang, J.-P. Guo, S.-H. Sun, Q. Sun, Y.-W. Zhao, Y.-F. Zhang, Z.-Y. Yu, C.-S. Li, Y. Sun, M.-M. Zhang, Y. Jiang, Optimized valence state of Co and Ni in high-entropy alloy for high active-stable oer. *Rare Met.* **42**, 3607–3613 (2023).

56. P. Yang, Y. An, C. Feng, Y. Liu, S. Liu, L. Gao, Y. Zhou, X. Li, P. Li, F. Zeng, Heterogeneous high-entropy catalyst nanoparticles for oxygen evolution reaction: Impact of oxygen and fluorine introduction. *Int. J. Hydrogen Energy* **51**, 1218–1228 (2024).

57. G. Raj, R. Nandan, K. Kumar, D. B. Gorle, A. B. Mallya, S. M. Osman, J. Na, Y. Yamauchi, K. K. Nanda, High entropy alloying strategy for accomplishing quintuple-nanoparticles grafted carbon towards exceptional high-performance overall seawater splitting. *Mater. Horiz.* **10**, 5032–5044 (2023).

58. J. X. Yang, B. H. Dai, C. Y. Chiang, I. C. Chiu, C. W. Pao, S. Y. Lu, I. Y. Tsao, S. T. Lin, C. T. Chiu, J. W. Yeh, P. C. Chang, W. H. Hung, Rapid fabrication of high-entropy ceramic nanomaterials for catalytic reactions. *ACS Nano* **15**, 12324–12333 (2021).

59. H. Y. Wang, R. Wei, X. M. Li, X. L. Ma, X. G. Hao, G. Q. Guan, Nanostructured amorphous $Fe_{29}Co_{27}Ni_{23}Si_9B_{12}$ high-entropy-alloy: An efficient electrocatalyst for oxygen evolution reaction. *J. Mater. Sci. Technol.* **68**, 191–198 (2021).

60. S. Q. Zhao, H. Y. Wu, R. Yin, X. N. Wang, H. Z. Zhong, Q. Fu, W. J. Wan, T. Cheng, Y. Shi, G. X. Cai, C. Z. Jiang, F. Ren, Preparation and electrocatalytic properties of $(FeCrCoNiAl_{0.1})O_x$ high-entropy oxide and NiCo-$(FeCrCoNiAl_{0.1})O_x$ heterojunction films. *J. Alloys Compd.* **868**, 159108 (2021).

61. P. Y. Ma, S. C. Zhang, M. T. Zhang, J. F. Gu, L. Zhang, Y. C. Sun, W. Ji, Z. Y. Fu, Hydroxylated high-entropy alloy as highly efficient catalyst for electrochemical oxygen evolution reaction. *Sci. China Mater.* **63**, 2613–2619 (2020).

62. K. Z. Gu, X. Y. Zhu, D. D. Wang, N. N. Zhang, G. Huang, W. Li, P. Long, J. Tian, Y. Q. Zou, Y. Y. Wang, R. Chen, S. Y. Wang, Ultrathin defective high-entropy layered double hydroxides for electrochemical water oxidation. *J. Energy Chem.* **60**, 121–126 (2021).

63. K. Huang, B. Zhang, J. Wu, T. Zhang, D. Peng, X. Cao, Z. Zhang, Z. Li, Y. Huang, Exploring the impact of atomic lattice deformation on oxygen evolution reactions based on a sub-5 nm pure face-centred cubic high-entropy alloy electrocatalyst. *J. Mater. Chem. A* **8**, 11938–11947 (2020).

64. Z. Chen, J. Wen, C. Wang, X. Kang, Convex cube-shaped $Pt_{34}Fe_5Ni_2Cu_{31}Mo_9Ru$ high entropy alloy catalysts toward high-performance multifunctional electrocatalysis. *Small* **4255**, 2204255 (2022).

65. Y. M. Zhang, J. L. Kang, H. A. Xie, H. X. Yin, Z. J. Zhang, E. Z. Liu, L. Y. Ma, B. Chen, J. W. Sha, L. H. Qian, W. B. Hu, C. N. He, N. Q. Zhao, Boosting the oxygen evolution of high-entropy (Oxy)hydroxide epitaxially grown on high entropy alloy by lattice oxygen activation. *Appl. Catal. B. Environ.* **341**, 123331 (2024).

66. J. H. Cha, S. H. Cho, D. H. Kim, D. Jeon, S. Park, J. W. Jung, I. Kim, S. Y. Choi, Flash-thermal shock synthesis of high-entropy alloys toward high-performance water splitting. *Adv. Mater.* **35**, 202305222 (2023).

67. A. N. Nguyen, N. M. Tran, H. Yoo, Direct growth and post-treatment of zeolitic imidazolate framework-67 on carbon paper: An effective and stable electrode system for electrocatalytic reactions. *J. Mater. Chem. A* **10**, 20770–20778 (2022).

68. Y. Li, Q. Zhang, X. Zhao, H. Wu, X. Wang, Y. Zeng, Q. Chen, M. Chen, P. Liu, Vapor phase dealloying derived nanoporous Co@CoO/RuO_2 composites for efficient and durable oxygen evolution reaction. *Adv. Funct. Mater.* **33**, 202214124 (2023).

69. W. Zhang, M. Niu, J. Yu, S. Li, Y. Wang, K. Zhou, Mechanochemical post-synthesis of metal-organic framework-based pre-electrocatalysts with surface Fe-O-Ni/Co bonding for highly efficient oxygen evolution. *Adv. Funct. Mater.* **33**, 202302014 (2023).

70. Y. F. Cui, S. D. Jiang, Q. Fu, R. Wang, P. Xu, Y. Sui, X. J. Wang, Z. L. Ning, J. F. Sun, X. Sun, A. Nikiforov, B. Song, Cost-effective high entropy core–shell fiber for stable oxygen evolution reaction at 2 A cm^{-2}. *Adv. Funct. Mater.* **33**, 202306889 (2023).

71. K. Yu, H. Yang, H. Zhang, H. Huang, Z. Wang, Z. Kang, Y. Liu, P. W. Menezes, Z. Chen, Immobilization of oxyanions on the reconstructed heterostructure evolved from a bimetallic oxysulfide for the promotion of oxygen evolution reaction. *Nanomicro. Lett.* **15**, 186 (2023).

72. M. Li, X. Wang, K. Liu, Z. Zhu, H. Guo, M. Li, H. Du, D. Sun, H. Li, K. Huang, Y. Tang, G. Fu, Ce-induced differentiated regulation of Co sites via gradient orbital coupling for bifunctional water-splitting reactions. *Adv. Energy Mater.* **13**, 202301162 (2023).

73. S. Wang, W. Huo, H. Feng, Z. Xie, J. K. Shang, E. V. Formo, P. H. C. Camargo, F. Fang, J. Jiang, Enhancing oxygen evolution reaction performance in prussian blue analogues: Triple-play of metal exsolution, hollow interiors, and anionic regulation. *Adv. Mater.* **35**, e2304494 (2023).
